# Anti-fungal bioactive terpenoids in the bioenergy crop switchgrass (*Panicum virgatum*) may contribute to ecotype-specific microbiome composition

**DOI:** 10.1038/s42003-023-05290-3

**Published:** 2023-09-07

**Authors:** Xingxing Li, Ming-Yi Chou, Gregory M. Bonito, Robert L. Last

**Affiliations:** 1 DOE Great Lakes Bioenergy Research Center, Michigan State University, East Lansing, MI 48824 USA; 2 Department of Biochemistry and Molecular Biology, Michigan State University, East Lansing, MI 48824 USA; 3 Department of Plant, Soil and Microbial Sciences, Michigan State University, East Lansing, MI 48824 USA; 4 Department Plant Biology, Michigan State University, East Lansing, MI 48824 USA; 5 Present Address: Department of Plant Biology, Rutgers University, New Brunswick, NJ 08901 USA

**Keywords:** Plant sciences, Biofuels, Fungi

## Abstract

Plant derived bioactive small molecules have attracted attention of scientists across fundamental and applied scientific disciplines. We seek to understand the influence of these phytochemicals on rhizosphere and root-associated fungi. We hypothesize that – consistent with accumulating evidence that switchgrass genotype impacts microbiome assembly – differential terpenoid accumulation contributes to switchgrass ecotype-specific microbiome composition. An initial in vitro Petri plate-based disc diffusion screen of 18 switchgrass root derived fungal isolates revealed differential responses to upland- and lowland-isolated metabolites. To identify specific fungal growth-modulating metabolites, we tested fractions from root extracts on three ecologically important fungal isolates – *Linnemania elongata, Trichoderma* sp. and *Fusarium* sp. Saponins and diterpenoids were identified as the most prominent antifungal metabolites. Finally, analysis of liquid chromatography-purified terpenoids revealed fungal inhibition structure – activity relationships (SAR). Saponin antifungal activity was primarily determined by the number of sugar moieties – saponins glycosylated at a single core position were inhibitory whereas saponins glycosylated at two core positions were inactive. Saponin core hydroxylation and acetylation were also associated with reduced activity. Diterpenoid activity required the presence of an intact furan ring for strong fungal growth inhibition. These results inform future breeding and biotechnology strategies for crop protection with reduced pesticide application.

## Abstract (web summary)

Ecotype-specific steroidal saponins and diterpenoids from *Panicum virgatum* (switchgrass) influence growth inhibition of fungal species previously associated with the rhizosphere of cultivars of this plant.

## Introduction

Renewable energy produced from plant biomass grown with ecologically sound practices can contribute to a more secure, environmentally sustainable and economically stable future^1^. Switchgrass (*Panicum virgatum*; Poaceae) has attracted attention as a biofuel feedstock because it is a perennial C4 photosynthetic plant with high cellulosic content, which thrives on land of little or no agricultural value^2,3^. Switchgrass also exhibits substantial resistance to pests and diseases^3,4^. As climate change is predicted to cause shifts in pest and microbial disease pressure that would endanger biomass production^5^, there is increasing opportunity to breed or metabolically engineer disease-resistant varieties. Unlike domesticated crops, switchgrass retains tremendous phenotypic diversity, including in-plant protective specialized metabolites^3,6,7^.

The interactions of plants with pathogenic and beneficial microbes are determined by many factors, including the production of specialized metabolites, whose synthesis and structures vary across the plant kingdom^8^. Terpenoids are an excellent example as they are the largest characterized plant natural product family^9^. The protective roles of terpenoid specialized metabolites have been extensively explored in the model plant, *Arabidopsis thaliana*, and major Poaceae crops. These include directly inhibiting microbial pathogen growth as toxins and priming plant defense. For example, the nonvolatile avenacin triterpenoid saponins protect oats from the fungal pathogen, *Gaeumannomyces tritici*, which causes ‘take-all’ disease^10–12^. In addition, the labdane diterpenoids that are broadly found in rice, wheat and maize have demonstrated defensive roles as antimicrobial phytoalexins^13–16^. Finally, C_10_ monoterpene and C_15_ sesquiterpene volatiles induce systemic acquired resistance within and between plants in *Arabidopsis*^17,18^.

The microbiome has demonstrable impact on plant growth and can act as an extension of the plant immune system^19^. Plants can recruit specific strains of microbes to help defend against pathogens through plant-microbe coevolution. At a community level, studies showed plant associated microbiome composition plays a crucial role in determining disease incidence and severity^20,21^. Manipulation of microbiomes was demonstrated to be a viable approach for plant disease control via microbe-mediated pathogen suppression^22^ or plant immune system priming^23^. Rhizosphere microbial compositions and functions are influenced by multiple factors, such as abiotic conditions^24,25^, plant species^26,27^, and plant genotypes^28–31^. Among these, variations in metabolites of plant above- and below-ground tissues as well as root exudates have been implicated in microbiome function^32–35^.

Species- or genotype-specific specialized metabolites play roles in mediating rhizosphere microbial interactions. For example, the secretion of coumarin and benzoxazinoid specialized metabolites from the root shape the maize rhizosphere microbiomes^36,37^. There is increasing evidence suggesting that switchgrass genotype influences the rhizosphere microbiome^28,38^. Our working hypothesis is that genotype-specific differences in root-specialized metabolites influence the growth of rhizospheric fungi.

As part of a larger effort to develop switchgrass varieties with high yield and low environmental impacts, we are engaged in studies of switchgrass metabolome screening by liquid chromatography-mass spectrometry (LC-MS) and other analytical chemistry methods. We previously identified close to 100 terpenoid metabolites, including saponins, diterpenes and sesquiterpenes, in the northern upland and southern lowland switchgrass ecotypes^6,39^. We demonstrated specific differences in metabolites across the terpenoid classes driven by ecotype, tissue/developmental stage and soil moisture, raising questions about their possible biological functions. Filling this knowledge gap will help lead to rational breeding of highly productive bioenergy crops with improved microbiome traits.

**Fig. 1:**
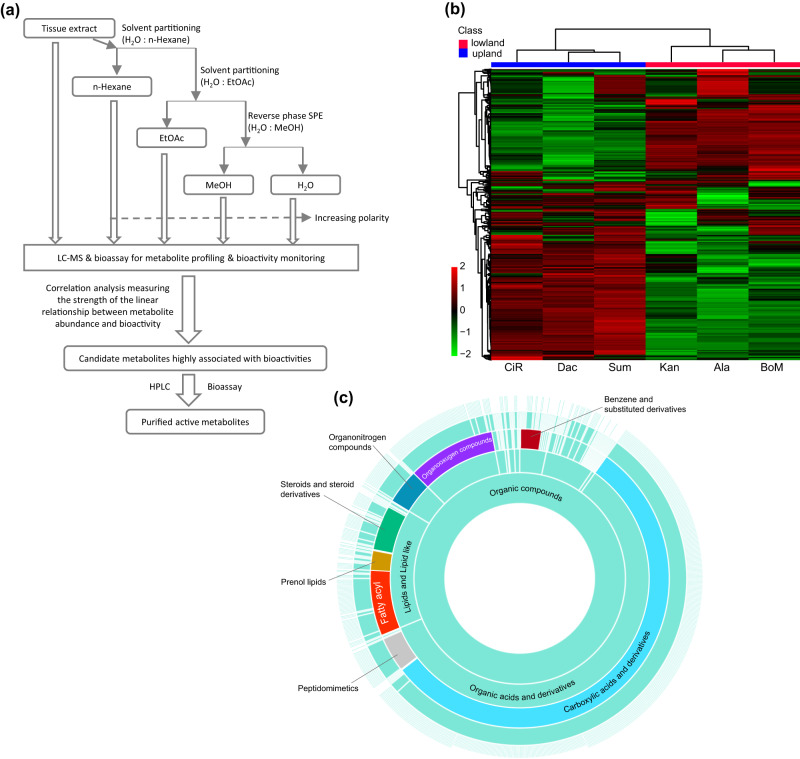
Workflow for the bioassay-guided identification of bioactive switchgrass metabolites and global view of switchgrass root tissue metabolomes revealed by untargeted metabolomics. **a** A workflow for isolation of bioactive compounds from switchgrass modified from Fig. 2 from Ebada et al.^75^. **b** Heatmap generated using Euclidean correlation-based clustering (algorithm: Ward) of Log_10_ Fold Change (FC) of the 1777 metabolite features identified in the root extracts of six switchgrass cultivars, with normalized abundance ≥ 500 arbitrary unit. Heatmap scale shows FC values. **c** Sunburst plot showing the CANOPUS classifications^40^ for switchgrass metabolites with *m/z* values ≤ 850 based on MS/MS spectra of the metabolite features. Inner rings represent parental classes of outer rings (from inside to outside: kingdom—super class—class—subclass—level5). On the compound class level, the eight largest classes are colored and labeled.

Here, we focused on abundant terpenoids that are dramatically different in root metabolomes among switchgrass ecotypes. We asked whether fungi isolated from switchgrass microbiomes are differentially sensitive to these terpenoids. We first showed that the upland and lowland switchgrass root extracts differentially inhibited the growth of 18 switchgrass rhizosphere fungi. Fungal bioactivity-guided metabolite fractionation (Fig. 1a) revealed that fractions containing saponins and diterpenoids inhibit fungal growth. The hypothesis was tested and confirmed through fungal bioassays using a set of high-performance liquid chromatography (HPLC) fractions containing specific steroidal saponins and diterpenoids. Together, our results revealed that terpenoids are active against diverse fungi in the switchgrass root microbiome and indicate that these bioactive natural products are candidates as mediators of switchgrass ecotype-specific microbiome recruitment.

## Results

### Terpenoids are ecotype-specific root metabolites

We followed up on previous observations that switchgrass root specialized metabolite profiles differ between upland and lowland ecotypes. We reasoned that a better annotated switchgrass metabolome would help to identify correlations between metabolites and antifungal bioactivities of root extracts from different switchgrass ecotypes/genotypes^6^. After extracting metabolites from large amounts of growth chamber grown root tissue biomass from the three upland and three lowland switchgrass cultivars (Methods), we evaluated the metabolites by LC-MS. Euclidean correlation-based clustering separated the three upland and lowland cultivars, confirming that these extracts have dramatically different metabolite constituents (Fig. 1b, Supplementary Data 1). Analysis of this untargeted metabolomics dataset using CANOPUS (class assignment and ontology prediction using mass spectrometry), a computational method for systematic compound class annotation^40^, assigned chemical classes to the features with *m/z* values ≤ 850 (Methods, Supplementary Data 2). This yielded a better understanding of the chemical composition of switchgrass root tissues, compared to annotating the features based on their relative mass defect^6^. Approximately half of the annotated features were carboxylic acids and derivatives, ~14% lipids and lipid derived metabolites and ~10% organooxygen compounds, which are mostly carbohydrates and carbohydrate conjugates (Fig. 1c, Supplementary Data 2).

**Fig. 2:**
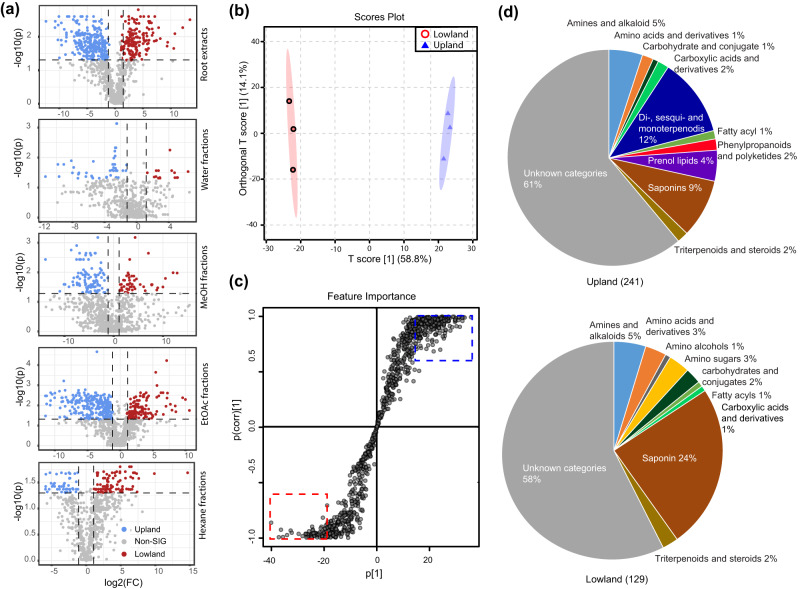
Comparative metabolomics of the upland and lowland switchgrass. **a** Volcano plots showing the results of significance analysis (cutoff threshold: FDR adjusted *p* ≤ 0.05; fold changes ≥ 2) that were used to identify the significant different (SIG) metabolites between the upland and lowland switchgrass ecotypes in the whole root extracts, water, methanol, EtOAc and hexane fractions, respectively. *n* = 3 samples each. **b** OPLS-DA scores plot of upland (blue triangles) and lowland (red circles) switchgrass whole root samples based on the normalized abundances (peaks areas) of 1777 metabolite features. **c** S-plot corresponding to the OPLS-DA model used to characterize the differentially accumulated features (DAFs) associated with upland (blue dotted rectangle) and lowland (red dotted rectangle) root samples. Cutoff values: covariance |*p*| ≥ 0.6 and correlation |p (corr)| ≥ 20 for whole root extracts. **d** Annotated compound classes for the identified DAFs in upland and lowland switchgrass root. Percentages for the shares within each pie chart, while the total numbers of DAFs are shown in parentheses. See the columns D, E and F of Supplementary Data 3, 4 for the feature annotations.

To identify the major metabolite classes contributing to the divergent switchgrass root metabolomes, we fractioned the extracts by polarity using two-phase separations with water/hexane followed by water/ethyl acetate (EtOAc) and then C18 resin solid phase extraction (SPE) eluted with water/methanol (Fig. 1a). The metabolite fold-change revealed that the medium- and low-polarity metabolites contribute to the overall profile divergence more than do those small molecules of high-polarity (Fig. 2a). Next, we performed Orthogonal Projections to Latent Structures Discriminant Analysis (OPLS-DA)^41^, to identify the ecotype-specific differentially accumulating features (DAFs) that drive the metabolite profile separation in each fraction (Fig. 2b, Fig. S1a–d). The analysis generated an S-plot from which the DAFs with high reliability and magnitude were selected according to a defined threshold (Fig. 2c, Fig S1e–h): 241 and 129 of the most highly weighted DAFs were identified for upland and lowland ecotypes, respectively (summarized in Fig. 2d from the annotation provided in Supplementary Data 3, 4). Using a combination of CANOPUS, LC-MS databases and manual annotation (Methods), we classified 39% and 43% of the upland and lowland DAFs into 10 chemical categories (Fig. 2d, Supplementary Data 3, 4). Notably, in the upland ecotype, 12% of DAFs were classified as C20 di-, C15 sesqui-, and C10 monoterpenoids, followed by 9% of DAFs grouped into the saponin class (C27-C30 core glycosylated triterpenoids and steroids) (Fig. 2d, Supplementary Data 3, 4). In strong contrast, 24% of lowland DAFs were classified as saponins while none were annotated as di-, sesqui- or monoterpenoids (Fig. 2d, Supplementary Data 3, 4).

### Analysis of antifungal activities from switchgrass root tissues

**Fig. 3:**
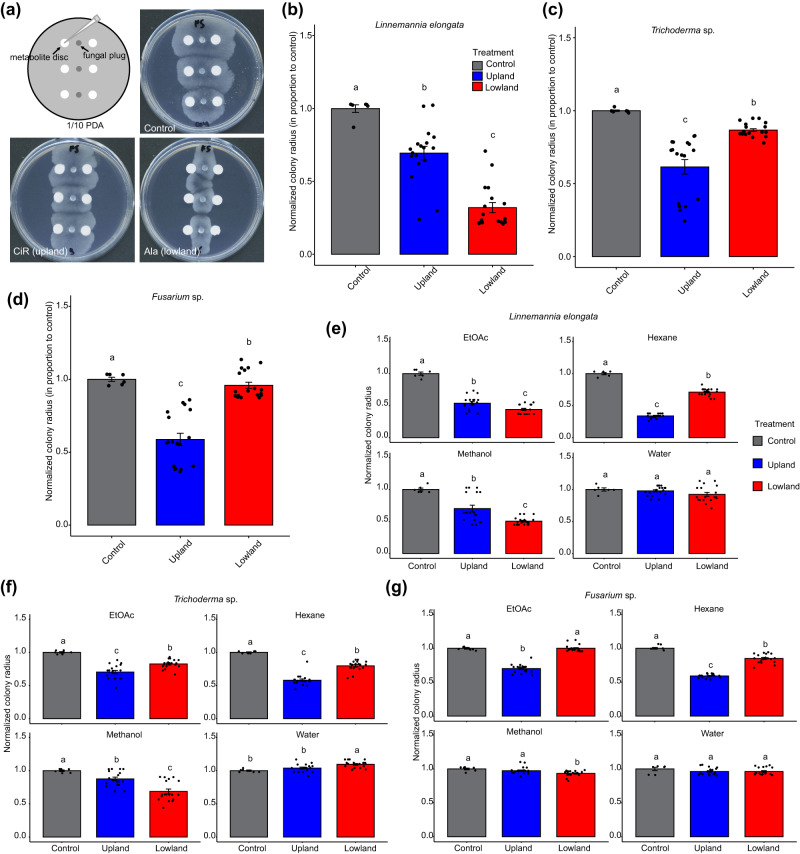
Extracts and fractions of upland and lowland switchgrass root tissues exhibit differential growth inhibition of the tested fungi. **a** Images of *L. elongata* colonies growing on the control (upper right), upland (lower left) and lowland (lower right) switchgrass root extracts in a disc diffusion assay. The fungal hyphae were inoculated along the middle vertical line at three positions; filter paper discs containing the plant extracts in ethanol were placed on both sides of each inoculation site (upper left; see Methods for more details). **b**–**d** Root extracts from individual upland switchgrass cultivars—Dacotah, Summer and Cave-in-Rock—and lowland cultivars—Alamo, Kanlow, and BoMaster–were used to screen against *L. elongata*, *Trichoderma* sp. and *Fusarium* sp., respectively, at a (w/v) concentration of 50 mg/mL in 70% ethanol. 70% ethanol was used as the negative control. The colony radiuses from the experimental groups were normalized to the ethanol only control colony radiuses. *n* = 3 (cultivars) × 6 (replicates per cultivar) = 18 (total replicates). **e**–**g** Growth inhibition of different metabolite fractions from the individual upland and lowland switchgrass cultivars, used at 10 mg/mL, against *L. elongata*, *Trichoderma* sp. and *Fusarium* sp. Distilled water, 80% methanol, EtOAc and hexane were used as the negative control correspondingly. The colony radiuses from the experimental groups were normalized to the control colonies. *n* = 3 (cultivars) × 6 (replicates per cultivar) = 18.

The ecotype-specific differences in metabolite accumulation provided an opportunity to evaluate responses of epiphytic and endophytic fungi isolated from field-grown switchgrass roots to extracts with unique compositions. Disc diffusion assays were set up using mycelium inocula of 18 different fungi (Table S1), which were isolated from switchgrass roots as previously published^28^. These isolates were selected to represent fungal phylogenetic diversity and abundance across switchgrass ecotypes. These were treated using filter paper discs containing upland or lowland switchgrass root extracts or solvent controls (Fig. 3a).

The results showed that most of the tested fungi were inhibited in growth by upland and/or lowland root tissue extracts. Of note, the upland extracts inhibited more (17 out of 18) fungi than did the lowland extracts (11 out of 18, Figs. 3b–d and S2). The growth of only one fungus (*Myrothecium* spp.) was promoted by the extracts of both ecotypes (Fig. S2). We were particularly interested in the fungi that differentially responded to the upland and lowland root tissue extracts; thus, we chose *Linnemannia elongata* (GLBRC 635), *Trichoderma* sp. (GLBRC 496) and *Fusarium* sp. (GLBRC 120) for detailed analysis. While extracts of both ecotypes suppressed the *L. elongata* growth, lowland ecotype cultivar extracts inhibited its growth more effectively when used at a comparable concentration (Fig. 3b). In contrast, *Trichoderma* sp. and *Fusarium* sp. growth was strongly suppressed by the upland cultivar extracts, while lowland extracts were minimally effective (Fig. 3c, d).

We next asked whether the differences in fungal growth resulted from responses to the ecotype differentially accumulated metabolites. *Linnemannia elongata*, *Trichoderma* sp. and *Fusarium* sp. were first treated with fractions of upland and lowland switchgrass root tissue extracts that differed by polarity and other chemical properties (Fig. 1a). Differential *L. elongata* growth inhibition was observed by EtOAc and hexane partitioned metabolites as well as SPE methanol fractions of the two ecotypes. The lowland EtOAc and methanol fractions were more suppressive than those of the upland cultivars, whereas the upland hexane fraction was more suppressive than the lowland one (Fig. 3e). The *Trichoderma* sp. isolate behaved similarly to *L. elongata* except that its growth was inhibited to a greater extent by the upland EtOAc fraction (Fig. 3f). Differential suppression by the methanol, EtOAc and hexane fractions was also observed for *Fusarium* sp.; for this isolate, the lowland methanol fraction was more suppressive than the upland methanol fraction. In contrast, the upland EtOAc and hexane fractions were more suppressive than the corresponding lowland fractions (Fig. 3g). In contrast to the varied antifungal activities of the metabolites that partitioned into the various organic phases, none of the high-polarity metabolites enriched in the water phase inhibited the tested fungi. *Trichoderma* sp. growth was promoted slightly by the lowland water phase (Fig. 3e–g).

**Fig. 4:**
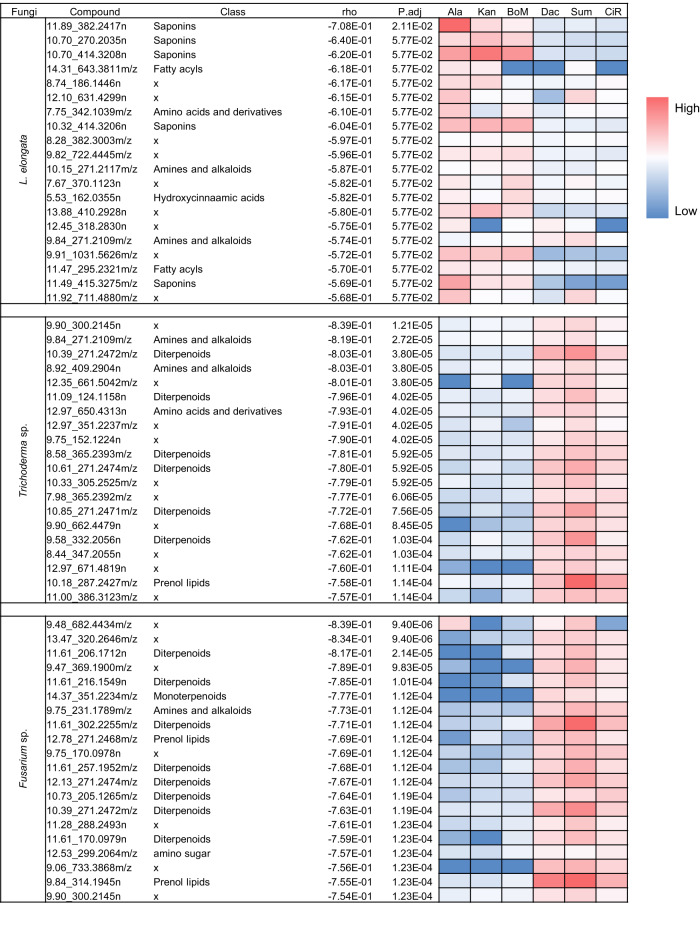
The top 20 most potent antifungal metabolites in switchgrass root tissue extracts revealed by correlation analysis. Spearman’s rank correlation coefficient was calculated to show the strength of relationship between the feature abundance and fungal growth. The coefficient, ‘rho’, has a value between 1 and −1. The features that are most negatively correlated to fungal growth are the best candidates for those contributing most to the antifungal activities of the switchgrass root extracts. The significance of the Spearman’s rank correlation coefficients was also calculated, reflected by the FDR-adjusted *p*-values. Compound classes were predicted by either CANOPUS or database searching through the Progenesis QI. ‘x’ means no compound class information associated with the feature. Features that are of high abundance in the switchgrass roots are in red, those that are of low abundance are in blue. Lowland ecotypes: Ala Alamo, Kan Kanlow, BoM BoMaster. Upland ecotypes: Dac Dacotah, Sum Summer, CiR Cave-in-Rock.

To elucidate candidate bioactive metabolites enriched in the methanol, EtOAc or hexane fractions, we performed Spearman’s rank correlation analysis to associate the MS features to antifungal performances in different root-derived fractions (Fig. 1a). Remarkably, terpenoid specialized metabolites were highly correlated with the antifungal properties of switchgrass root extracts and fractions, revealed by the annotations of 20 MS features most highly correlated with inhibition of each of the three fungi. Five of the 20 features most inhibitory to *L. elongata* were annotated as saponins (Fig. 4). For example, the top correlated feature was a previously unidentified steroidal saponin glycosylated at a single core position whose MS/MS spectra revealed evidence for the presence of two hexoses one of which is acetylated (Fig. S3, Supplementary Data 5). In contrast, diterpenoids were shown to be among six and eight of the most highly inhibitory metabolite features against *Trichoderma* sp. and *Fusarium* sp., respectively (Fig. 4). Moreover, the candidate bioactive saponins and diterpenoids differed in root accumulation between the two switchgrass ecotypes; the saponins accumulated at higher levels in the lowland ecotype whereas the diterpenoids were more abundant in the upland root (Fig. 4). These correlative results led us to test the hypothesis that the contrasting antifungal properties of switchgrass root extracts are derived from these terpenoids.

### Saponin and diterpenoid antifungal properties and structure–activity relationships (SARs)

**Fig. 5:**
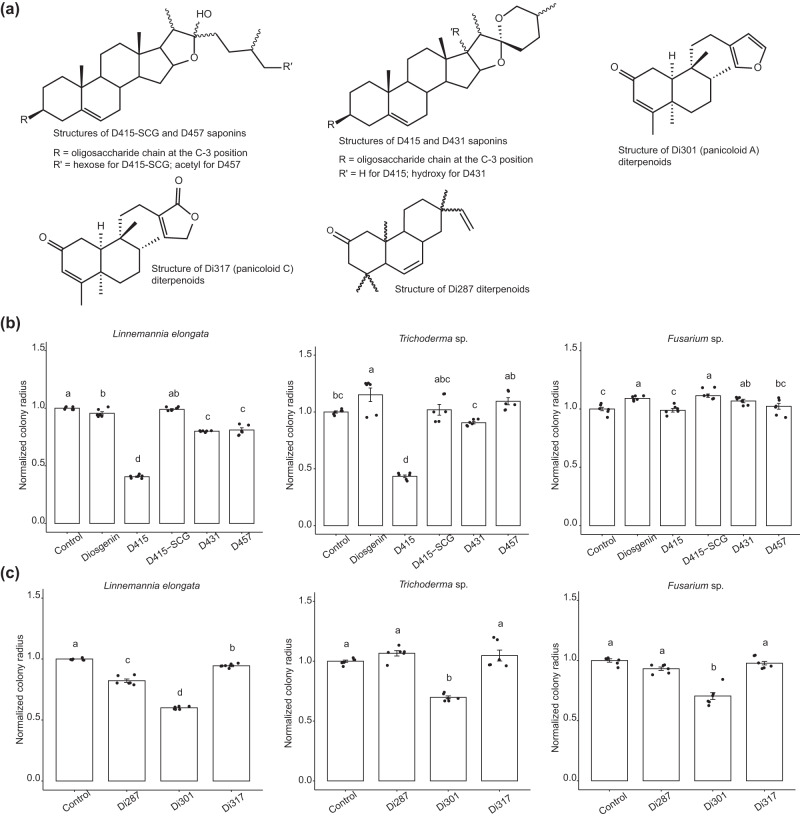
Efficacy of saponins and diterpenoids against the switchgrass rhizosphere fungal growth revealing structure-activity relationships. **a** The structures of HPLC purified switchgrass saponins and diterpenoids used in the bioassays. The structures of the saponins D415-SCG, D415, D431, and D457, and the diterpenoids, Di301 and Di317, were previously elucidated by LC-MS/MS and NMR^6,39^. NMR data supporting the structure of diterpenoid Di287 can be accessed in the supplementary files (Figs. S4–12, Table S2). **b** Bioactivities of different saponin types were screened against *L. elongata*, *Trichoderma* sp. and *Fusarium* sp. in the disc diffusion assay described in Fig. 3a. 50% methanol was used as the negative control. **c** Bioactivities of different diterpenoids were screened against the three tested fungi in a disc diffusion assay. Pure methanol was used as the negative control. For both the saponin and diterpenoid compounds, the tested concentration was 1 mg/mL. The colony radiuses from the experimental groups were normalized to those of methanol control colonies, which were set as the scale 1 on the bar plots (*n* = 6).

To test this hypothesis, we further fractionated several structurally distinct saponins and diterpenoids from root tissues of upland or lowland switchgrass using reverse phase semi-preparative HPLC, testing their antifungal activities using the disc diffusion assay. While the resultant HPLC samples contained multiple saponins or diterpenoids, they shared the same terpene cores with differences in modifications and stereo/structural chemistry (Supplementary Data 5). Taken together, these fractions included four classes of saponins (D415, D415-SCG, D431, and D457), which differ in their sapogenin aglycones and numbers of sugar chains, as well as three classes of diterpenoids (Di287, Di301 and Di317) that vary in their terpene backbones (Fig. 5a, Supplementary Data 5).

For the saponins, we examined the activity of commercial diosgenin (a sugarless, or aglycone, sapogenin core for all the currently identified switchgrass saponins) against *L. elongata*, *Trichoderma* sp. and *Fusarium* sp. isolates. Consistent with the hypothesis that the sugar moieties are required for saponin anti-microbial activities^42^, we found no inhibitory effect with diosgenin. In contrast, we observed inhibition of *L. elongata* and *Trichoderma* sp. with the D415 saponins glycosylated at a single core position. Interestingly, the D415-SCG saponins which are glycosylated at two core positions were shown not to be inhibitory, revealing that the presence of an additional sugar moiety at the saponin backbone C-26 position resulted in loss of detectable antifungal activity (Fig. 5b). Structural specificity for fungal growth inhibition by the saponins glycosylated at a single core position was supported by the observation that D431 and D457 (with either the C-17 hydroxylation or sidechain C-26 acetylation, respectively) showed dramatically decreased bioactivities compared to the unmodified D415 (Fig. 5b). Diterpenoids also exhibited differences in SAR. All three fungi were inhibited by the Di301 panicoloid A furanoditerpenoids^39^ (Fig. 5c). In contrast, only *L. elongata* was also sensitive to the other two tested diterpenoids. One of these is the Di317 furanoditerpenoid panicoloid C^39^ whose furan ring is oxidized to an unsaturated lactone (furanone) (Fig. 5c). The other one, Di287, is a previously structurally uncharacterized and drought-inducible diterpenoid^39^. We used a set of one- and two-dimensional NMR experiments to resolve its structure (Methods), revealing that this metabolite as a pimarane diterpenoid without a furan ring (Figs. S4–12, Table S2).

The effect of the saponins D415-SCG and D415 and the diterpenoids Di301 and Di317 on the growth of *L. elongata* was examined in liquid cultures at three concentrations (0.1, 0.005, and 0.00025 mg/mL). Those results were generally consistent with the agar-based growth assay. The D415 saponin glycosylated at a single core position clearly inhibited fungal growth in a dose-dependent manner in two independent tests, while the growth inhibition by the D415-SCG saponin glycosylated at two core positions was only seen at the highest concentration in one test (Fig. S13a). The diterpenoids Di301 and Di317 inhibited fungal growth at the highest concentrations in the first test and showed dose-dependent inhibition in the second test (Fig. S13b). Together, these results revealed antifungal activity differences among the structurally distinct switchgrass terpenoids.

## Discussion

Switchgrass, an important constituent of the North American tallgrass prairie ecosystem, is valued as a biofuel crop for its high net energy yield and relatively low economic and environmental costs associated with irrigation, fertilizers and pesticides^1,3^. We are interested in the possible roles of specialized metabolites in mediating documented differences in a variety of biotic and abiotic stress tolerances between the northern upland and southern lowland ecotypes of this species^43–46^. Here, we tested the bioactivities of upland and lowland root metabolites on 18 switchgrass rhizosphere-associated fungal species, demonstrating that the steroidal saponins and diterpenoids influence ecotype-specific growth inhibition of these fungi.

Untargeted metabolomics revealed distinctive metabolomes for upland and lowland switchgrass roots ^6^. Classification of the differentially-accumulating mass features revealed that terpenoids are among the most prominent ecotype-specific metabolites. Notably, a variety of unglycosylated mono- (C10), sesqui- (C15) and diterpenoids (C20) were identified, accounting for 12% of upland switchgrass DAFs while none of these metabolites were identified among the lowland ecotype DAFs (Fig. 2d, Supplementary Data 3, 4). In contrast, saponins—glycosylated phytosteroids or triterpenoids containing backbones of 27–30 carbons—were enriched among the lowland switchgrass-specific metabolites. Specifically, 24% of lowland and 9% of upland ecotype DAFs were annotated as saponins (Fig. 2d, Supplementary Data 3, 4). Therefore, the metabolomics analysis suggested that diterpenoids and saponins are characteristic of upland and lowland switchgrass root metabolomes, respectively.

To evaluate the bioactivities of upland and lowland switchgrass root metabolites, we screened upland and lowland switchgrass root extracts for their growth impacts on 18 diverse fungal species previously isolated from switchgrass rhizosphere as epiphytes or endophytes^28^. These fungi were chosen based on their prevalence in switchgrass-associated environments and potential ecological roles^28^. Growth of 17 of 18 fungi was inhibited by the C10, C15 and C20 terpenoid-rich upland root extracts while saponin-dominated lowland root extracts suppressed the growth of 11/18 isolates. Sixteen of the 18 tested fungi (16/18) differentially responded to the upland and lowland root extracts (Figs. 3b–d and S2). Correlation analysis of these results led us to hypothesize that these fungi are more sensitive to unglycosylated terpenoids or saponins.

To pursue the hypothesis, we fractionated switchgrass root extracts based on polarity and screened representatives of three ecologically important fungal taxa, *L. elongata*, *Trichoderma* sp. and *Fusarium* sp., with these fractions. *Fusarium* is a prominent fungal genus in plant and soil environments^47,48^, and the genus includes plant pathogens of economic importance^49^, as well as rare documented cases of beneficial effects where non-pathogenic strains of *Fusarium* compete with pathogenic *Fusarium* in diseases such as *Fusarium* wilt^50^. *Trichoderma* isolates were documented to be beneficial, pathogenic, or commensal. As one of the most prevalent fungi in soil and plant environment^51^, they were also found to be plant symbionts that promote plant growth and defense^52–55^. However, a few species were also found to be pathogenic to plants^56,57^. *Linnemannia*, formerly classified as *Mortierella*, is closely associated with switchgrass roots^28,58^. Isolates of the species promoted growth of *Arabidopsis* and economically important crops such as *Citrullus lanatus*, *Zea mays*, *Solanum lycopersicum*, *Cucurbita* and *Calibrachoa* through inducing IAA production and facilitating P and Fe acquisition^59–63^.

We tested the bioactivities of upland and lowland switchgrass root fractions with different metabolite compositions against these three focal fungal species. Overall, we observed differential responses of these species to the saponin enriched methanol fraction and unglycosylated terpene enriched EtOAc/hexane fractions. For example, growth of *L. elongata* was more sensitive to the lowland methanol fraction whereas the upland EtOAc/hexane fractions inhibited *Trichoderma* sp. and *Fusarium* sp. (Fig. 3e–g). Furthermore, correlation analysis (Fig. 4) predicted that growth of the three focal species was most highly influenced by saponins and diterpenoids.

We tested the bioactivities of these terpenoids by using a set of structurally distinct steroidal saponins and diterpenoids purified from upland and lowland switchgrass roots (Fig. 5). This SAR analysis revealed that the saponin antifungal activity was primarily associated with the number of saponin sugar chains: our results revealed that the saponins glycosylated at a single core position were inhibitory whereas those glycosylated at two core positions were not (Fig. 5b). The C-3 sugar substitution seems to be necessary for the antifungal activities as the aglycone sapogenin, diosgenin, only minimally inhibited the growth of *L. elongata*, and even slightly promoted growth of *Trichoderma* sp. and *Fusarium* sp. (Fig. 5b). In addition, the C-17 hydroxylation and C-26 acetylation dramatically reduced the antifungal activities of saponins with a single sugar chain (Fig. 5b). A similar observation was reported for oat avenasides, where the 26-desgluco-avenacosides inhibited *Sordaria macrospora* growth, while the intact avenacosides showed no effect at the same or twofold higher concentration^64^. These results are consistent with the hypothesis that fungal growth inhibition by saponins acts by their hemolytic activity—the same molecular mechanism causing lysis of mammalian erythrocytes^42,65–67^. Such a mechanism requires both hydrophilic sugar chains and non-polar end of sapogenin core for the formation of aqueous pores leading to membrane perturbation^68–70^.

We purified and elucidated the structure for a novel pimarane type diterpenoid—Di287–from switchgrass roots. In contrast to the previously identified Panicoloid diterpenoids (Di301 and Di317)^39^, the NMR evidence indicated a lack of a furan moiety in the diterpene scaffold of Di287 (Figs. S4–12). We then explored SAR of three different types of switchgrass diterpenes—Di301, Di317, and Di287—with and without a furan moiety. In contrast to the strong structural specificity of saponins, all three types of diterpenoids had antifungal activities against *L. elongata* (Fig. 5c). Di301, containing a furan moiety in its side chain, exhibited the strongest growth inhibition. It also inhibited the growth of *Trichoderma* sp. and *Fusarium* sp., while Di287—lacking the furan moiety—and the oxidized furan ring containing Di317 did not (Fig. 5c). These observations indicate that a furan ring increases the switchgrass diterpenoid antifungal activities, while oxidation of this moiety results in lower potency. Regardless of the structural characteristics, significantly increased accumulations of all three diterpenoid types were observed in the roots of drought-stressed switchgrass^39^, suggesting potential functions of these structurally diverse terpenoids beyond their antifungal activities.

In conclusion, we demonstrated differential inhibition of ecologically important fungi isolated from the rhizosphere of the perennial bioenergy crop by saponins and diterpenoids—the metabolites preferentially accumulated in the upland and lowland switchgrass roots, respectively. Results of this study add to published work demonstrating that the antimicrobial properties of plant-specialized metabolites influence microbiome assembly in crops as diverse as *Panax notoginseng*^71^, *Camellia oleifera*^72^, *Lilium Oriental*^73^, and *Zea mays*^74^. Together with these previous studies, our results suggest that ecotypic differences in switchgrass terpene specialized metabolites might differentially influence soil fungal microbiome activities. This study sets the stage for further investigation of the in vivo roles of these specialized metabolites in assembling switchgrass-associated microbial communities and developing breeding strategies to facilitate biomass production and sustainable cropping system for switchgrass.

## Methods

### Plant materials

Seeds for the upland (Dacotah, Summer and Cave-in-Rock) and lowland (Alamo, Kanlow, and BoMaster) switchgrass were purchased from Native Connections (http://nativeconnections.net, Three Rivers, MI). The seeds were sowed into a 1:1 mixture of sand and vermiculite and grown for three months at 27 °C with 16 h light (cool white fluorescent light bulbs, 500 μE m^−2^ s^−1^) per day in a growth chamber. The relative humidity in the chamber was set to 53% without dehumidification.

### Metabolite extraction and fractionation

All chemicals were obtained from Sigma Aldrich (St. Louis, MO) unless otherwise specified. Switchgrass plants were pulled out of pots and cut ~1 cm above the rhizome to collect the root tissues. Attached sand and vermiculite were carefully washed from the root surface using deionized water and blotted dry by paper towels. 100–200 g fresh switchgrass root tissues were chilled in liquid nitrogen and powdered using mortar and pestle. Total metabolites were obtained by immediately adding 80% methanol to the powders at a tissue/solvent ratio 1:10 (w/v) and extracting (without stirring) at 4 °C overnight. The solvent was removed and the extraction was repeated with fresh 80% methanol at 4 °C overnight. The combined solvent was centrifuged at 4000 *g* for 15 min to remove the debris, pooled, concentrated using a rotary evaporator and then completely dried down in a SpeedVac concentrator (Thermo Scientific, Waltham, MA). The dried extracts were stored in a −80 °C freezer.

Sequential liquid-liquid phase partitioning and solid phase extraction (SPE) for the whole root extracts was carried out according to a published protocol^75^. Briefly, the dry extracts were dissolved in 100 mL water and phase partitioned against hexane at a 1:1 ratio in a separatory funnel. The water phase was next phase partitioned against EtOAc. Each of these liquid-liquid phase partitioning steps was done only once. After that, the resultant water phase was loaded to a 35 cc C18 SPE cartridge (Waters, Milford, MA). The cartridge was first washed using 3 × 20 mL of each 0%, 10%, and 20% methanol (in water). The flowthrough and wash were collected, pooled and considered as the final ‘water phase’ in this study. The cartridge was then eluted again using 3 × 20 mL of each 50%, 70%, 80%, and 90% methanol (in water). These washes were collected, pooled and called the ‘methanol phase’. All fractions were evaporated to dryness under vacuum using a SpeedVac and stored in the -80 freezer before bioassay.

### Compound purification

The methanol fraction and EtOAc/hexane fraction were used for steroidal saponin and diterpenoid isolation via reverse-phase HPLC, respectively. Steroidal saponins were purified from the methanol fraction of lowland switchgrass, Kanlow, root extracts, following the procedures previously described^6^. Diterpenoids were purified from the EtOAc and hexane fractions of upland switchgrass, Cave-in-Rock, root extracts. Diterpene purification was performed using a Waters 2795 pump/autosampler connected with LKB Superrac 2211 fraction collectorand a Waters Symmetry C18 HPLC column (100 Å, 5 µm, 4.6 mm × 150 mm). The water mobile phase was adjusted to PH 2.8 (Solvent A) and acetonitrile was used as the organic mobile phase (Solvent B). A 20-min linear gradient elution was used to purify all three diterpenoids, Di287, Di301, and Di317, starting from 1% B at 0 min, 40% B at 1.01 min and held at 40% between 1.01 and 3 min, linear increased to 90% at 15 min, 99% B at 15.01 min and held at 99% B between 15.01 and 18 min. The solvent flow rate and column temperature were set at 1.5 mL/min and 40 °C, respectively. Followed by a sample injection (100 µL), the 20-min LC gradient starts during which liquid droplets were collected, by using a fraction collector, every 10 secs as a single fraction. In the end, 120 fractions were obtained. The HPLC fractions containing the same targeted compounds, viewed by LC-MS, were pooled and dried down under vacuum in a SpeedVac.

### LC-MS metabolite analysis

For each LC-MS sample preparation, 500 mg fresh switchgrass root tissues were powered with liquid nitrogen using on a Mini G homogenizer (SPEX SamplePrep, Metuchen, NJ) and extracted in 5 mL 80% methanol containing 1 μM telmisartan internal standard and incubated for 16 h at 4°C (without stirring). Samples were centrifuged for 20 min at 4000 g and the supernatants transferred into 2-mL HPLC vials and stored in a −20 °C freezer prior to LC-MS analysis. The switchgrass metabolite separation and MS analysis were done using a reversed-phase UPLC BEH C18 (2.1 mm × 150 mm, 1.7 μm column, Waters) connected with an Electrospray Ionization—Quadrupole Time-of-Flight MS (ESI-QToF-MS, Waters). Both positive and negative mass spectral information were acquired under data-independent acquisition (DIA, MS^E^) and data-dependent acquisition (DDA, MS/MS) modes. The later method was specifically used for feature annotation purpose. All LC gradient and MS condition experimental parameters were as previously reported^6^.

### Untargeted metabolomics data processing

The raw MS data pre-treatments, including peak picking, adduct ion grouping, retention time (RT) alignment and lock mass correction, were performed in the Progenesis QI software (v3.0, Waters). By doing so, the metabolite features were identified, and each was defined by its RT and mass-to-charge (*m/z*) value. The intensities (abundances) of features were calculated from the peak area information, and then filtered to choose the ones with abundances ≥500 for the downstream statistical analysis. The parameters used with Progenesis processing were the same as described in the previous study^6^. The untargeted metabolomics feature annotation was achieved in two separate ways. First, online databases, including MassBank, MetaboLights, Plant Metabolic Network and PlantCyc were used to in silico search for the matching compounds deposited in the databases with the features identified from switchgrass, based on 10 ppm precursor tolerance, 95% isotope similarity and 10 ppm theoretical fragmentation pattern matching with fragment tolerance. Second, the MS/MS data acquired by DDA were used for chemical class predications for the untargeted metabolomics features using the CANOPUS (class assignment and ontology prediction using mass spectrometry) machine learning function^40^ built in the SIRIUS 4, which is a java-based software framework for the analysis of LC-MS/MS data of metabolites (https://bio.informatik.uni-jena.de/sirius/).

### NMR Spectroscopy

For NMR analysis of the Di287 diterpenoid, the HPLC purified sample was dried under vacuum and dissolved in deuterated chloroform (CDCl_3_). NMR spectra were acquired using a Bruker Avance NEO 600 MHz spectrometer (Bruker Biospin, Germany) operating at 600.13 MHz for proton experiments and equipped with a 5 mm nitrogen cryogenic HCN Prodigy probe and a SampleCase autosampler with sample cooling capability to 6 °C. For ^1^H NMR spectra, solvent suppression with shaped pulse program (wetdc) was used with a scan number (ns) of 64 at temperature 298 K with pulse width 8 µs and power 10.3 W. Acquisition time for each scan was 0.681 s with a 10 s delay time for a spectral width (sw) of 19.8 ppm. The magnetic field was locked to the solvent, CDCl_3_ and ^1^H spectra were calibrated using the residual solvent peaks. For the subsequent 1D and 2D NMR experiments, the spectra were collected using default Bruker pulse program jresqf for HOMO2DJ (ns = 16, sw = 19.8), zgpg30 for ^13^C (ns = 10,000, sw = 236), cosygppqf for COSY (ns = 8, sw = 13 for F1 & F2), hsqcedetgpsisp2.3 for HSQC (ns = 16, sw = 13 for F2 and 220 for F1), hmbcetgpl3nd for HMBC (ns = 32, sw = 13 for F2 and 220 for F1), h2bcetgpl3 for H2BC (ns = 32, sw = 15.1 for F2 and 180 for F1) and mlevphpp for TOCSY (ns =64, sw = 13 for F1 & F2) with pulse width 8 µs and power 10.3 W for ^1^H and pulse width 11.5 µs and power 152 W for ^13^C. All NMR spectra were acquired with Topspin 3.5.6. (Bruker) and analyzed using MestReNova 14.2.0 software (Mestrelab Research, Spain). Chemical shifts were normalized to the chloroform NMR solvent signals of ^1^H 7.26 *ppm* and ^13^C 77 *ppm*.

### Fungal bioassay

There were in total 18 fungal isolates (Table S1) used in this study which were obtained from switchgrass roots as previously described^28^. The 18 fungal isolates were selected to cover phylogenetic diversity, as well as being abundant across ecotypes based upon results obtained from a previous study^28^. The initial fungal growth assays were performed on all 18 isolates with bulk root extracts using a disc diffusion method. For root extracts from each switchgrass cultivar, six replicates were conducted on a 5 cm radius Petri dish containing 0.1 × PDA, comprised of potato dextrose broth mix (BD Diagnostics, Sparks, MD) at 2.4 g/L concentration, agar at 1.5% concentration amended with streptomycin and kanamycin at 50 µg/mL concentration. To commence the assay, 8 µL root extracts pre-dissolved in 70% ethanol at 50 mg/mL concentration was applied to each sterilized 0.6 cm radius round filter paper discs (Whatman 4, Cytiva, Maidstone, UK), and the solvent was allowed to evaporate in a biosafety hood for 1 hr before placing on the growth media. Fungi were grown on PDA, and 2 mm radius plugs were generated with a flame sterilized metal borer. These were used to inoculate the fungal growth assay by placing the plugs 1 cm away from the paper discs on each side. Fungal species-switchgrass cultivar growth assays were compared to a control that had paper discs with 70% ethanol followed by evaporation in a biosafety hood for 1 h. The Petri dishes were incubated under room temperature (~23 °C) and the colony horizontal growth from the center of the fungal plug to the rim of the mycelia was measured every other day.

From the initial 18 isolate screening, three isolates, *Trichoderma* sp. (GLBRC 497), *Fusarium* sp. (GLBRC 120) and *Linnemannia elongata* (GLBRC 635), with ecological importance were then chosen to proceed with the follow-up assays. The fungal growth assays on metabolite fractions from switchgrass root extracts were performed according to the above method except the paper discs were applied with metabolites dissolved in distilled water, 80% Methanol, EtOAc or hexane. The metabolite concentration (w/v) for each fraction was 10 mg/mL. The fungal growth was compared with a control grown with evaporated paper discs applied with the same solvent as the relevant fraction.

Liquid culture assays were set-up in pre-weighted 2 mL microcentrifuge tubes with 1.8 mL 0.1 x PDB each containing purified Di301, Di317, D415, or D415-SCG at concentrations of 0.1, 0.005, and 0.00025 mg/mL, and 20 μl of *L. elongata* hyphae lysate in six replicates. Extra no metabolite positive and 0.1X PDB only negative controls were prepared with six replications to compare with the treatment, respectively. The lysate of *L. elongata* hyphae was prepared by putting approximately 100 mg fresh PDB cultured *L. elongata* hyphae in 1.8 ml 0.1X PDB along with three 2 mm radius sterile metal beads, and vortexing at maximum speed for 3 min. Metal beads were removed after vortexing. The liquid culture assay was incubated on an orbital shaker at 100 RPM and 25 °C for 96 h before measuring the hyphae fresh weight. The hyphae fresh weights were measured by first centrifuging the 2 mL tubes containing the liquid fungal culture at 17, 000 *g* for 10 min at the room temperature to pellet down the hyphae. The supernatant was removed using a P1000 pipette. The resultant tubes with fungal hyphae were weighted on a XP26 high-performance analytical balance (Mettler-Toledo, Columbus, OH). The hyphae fresh weight was obtained by subtracting the weight of the empty 2 mL tube from the weight of the tube with the hyphae.

Measured fungal growth was normalized against the control to make all the metabolite-treated fungal growth relative to the growth of the control. All measurements from the disc diffusion and liquid culture assays were statistically analyzed and visualized with one-way ANOVA followed by Tukey’s test in R with packages “agricolae” and “ggplot2.”

### Statistics and reproducibility

Hierarchical Clustering Analysis (HCA), fold change analysis (volcano plot), Principal Component Analysis (PCA) and Orthogonal Projections to Latent Structures Discriminant Analysis (OPLS-DA) were done on the MetaboAnalyst 5.0 online tool platform. The *p*-values from all the significance analysis were adjusted by false discovery rate (FDR). An adjusted *p-*value ≤ 0.05 was considered statistically significant. The Spearman’s correlation test was conducted in R and *p*-values were adjusted with Benjamini and Hochberg false discovery rate correction method. For the statistical analysis of LC-MS-based metabolomics, the sample size is 30 (6 switchgrass cultivars × 5 treatments = 30). The number of replicates: three biological reps for both upland and lowland switchgrass ecotypes. In this case, one biological rep is a pool of 25 – 27 individual plants of a single switchgrass cultivar. For the statistical analysis of fungal bioassays testing the antifungal activities of switchgrass whole root extracts, the sample size is 756 (18 fungi × 6 switchgrass cultivars x 6 metabolite discs per plate + 18 fungi × 6 control disc per plate = 756. For the statistical analysis of fungal bioassays testing the antifungal activities of corresponding root extract fractions, the sample size is 486 (3 fungi × 6 switchgrass cultivars × 4 metabolite fractions × 6 metabolite discs per plate + 3 fungi × 4 metabolite fraction × 6 control disc per plate = 486). For the two cases above, the numbers of replicates are 18 for the experimental group and 6 for the control group. One replicate is a single disc loaded with either metabolite or control solvent on a single plate that was used to treat a particular fungus. For the statistical analysis of fungal bioassays testing the antifungal activities of purified compounds, the sample size is 180 (3 fungi × 8 compounds x 6 metabolite discs per plate + 3 fungi × 6 control disc per plate × 2 metabolite groups = 180). The numbers of replicates are 6 for both control and metabolite groups. One replicate is a single disc loaded with either metabolite or control solvent on a single plate that was used to treat a particular fungus.

### Reporting summary

Further information on research design is available in the Nature Portfolio Reporting Summary linked to this article.

### Supplementary information

Supplementary Figures and Tables

Description of Additional Supplementary Files

Supplementary Data S1-S13

Reporting Summary

## Data Availability

The raw data for LC-MS metabolomics and images of fungal bioassays are publicly available on DRYAD at 10.5061/dryad.stqjq2c7x. The rest of the data substantiating the conclusions of this study are available as Supplementary Figs. S1–S13, Supplementary Tables S1–S2 and Supplementary Data 1–5. Numerical source data for all fungal bioassay related graphs/charts can be found in Supplementary Data 6–13.
