# Supplementary material for: Anti-fungal bioactive terpenoids in the bioenergy crop switchgrass (Panicum virgatum) may contribute to ecotype-specific microbiome composition
Source: Commun Biol. 2023 Sep 7;6:917. doi: 10.1038/s42003-023-05290-3 (PMC10485007; doi:10.1038/s42003-023-05290-3)
Supplement: Supplementary file 1 — Supplementary Figures and Tables [file 42003_2023_5290_MOESM1_ESM.pdf]

Supplementary Figures for

Anti fungal bioactive terpenoids in the bioenergy crop switchgrass (*Panicum virgatum*) may contribute to ecotype-specfic mirobiome composition

Xingxing Li[1,2], Ming-Yi Chou[1,3,4], Gregory M. Bonito[1,3] and Robert L. Last[1,2,5 *]

[1]DOE Great Lakes Bioenergy Research Center, Michigan State University, East Lansing, MI, 48824 USA

[2]Department of Biochemistry and Molecular Biology, Michigan State University, East Lansing, MI, 48824 USA

[3]Department of Plant, Soil and Microbial Sciences, Michigan State University, East Lansing, MI, 48824 USA

[4]Present address: Department of Plant Biology, Rutgers University, New Brunswick, NJ, 08901

[5]Department Plant Biology, Michigan State University, East Lansing, MI, 48824 USA

Xingxing Li and Ming-Yi Chou contributed equally.

*Corresponding author: lastr@msu.edu

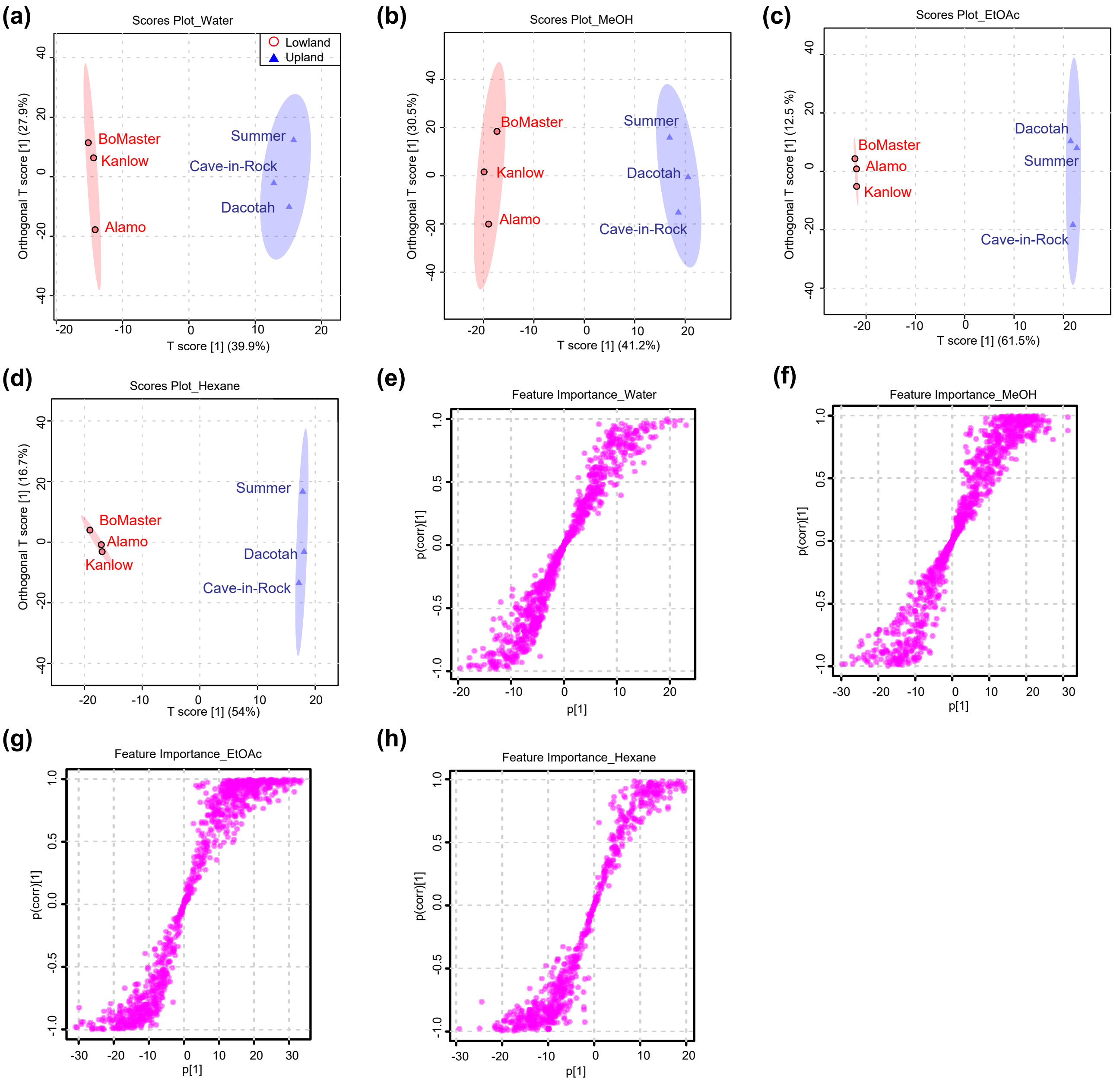

Figure S1. Differences in the metabolite profiles of upland and lowland switchgrass root fractions. (a) – (d) OPLS-DA score plots of upland (blue triangles) and lowland (red circles) switchgrass cultivar root fractions (water, MeOH, EtOAc and hexane) on the basis of normalized positive-mode LC-MS peak areas of 1777 metabolite features. (e) – (h) S-plots corresponding to the OPLS-DA models used to characterize the DAFs enriched in the upland and lowland root fractions. Cutoff values for the differentially accumulated features (DAFs): covariance $|p| \geq 0.6$ and correlation $|p\ (corr)| \geq 20$ for methanol and EtOA; covariance $|p| \geq 0.6$ and correlation $|p\ (corr)| \geq 10$ for water and hexane.

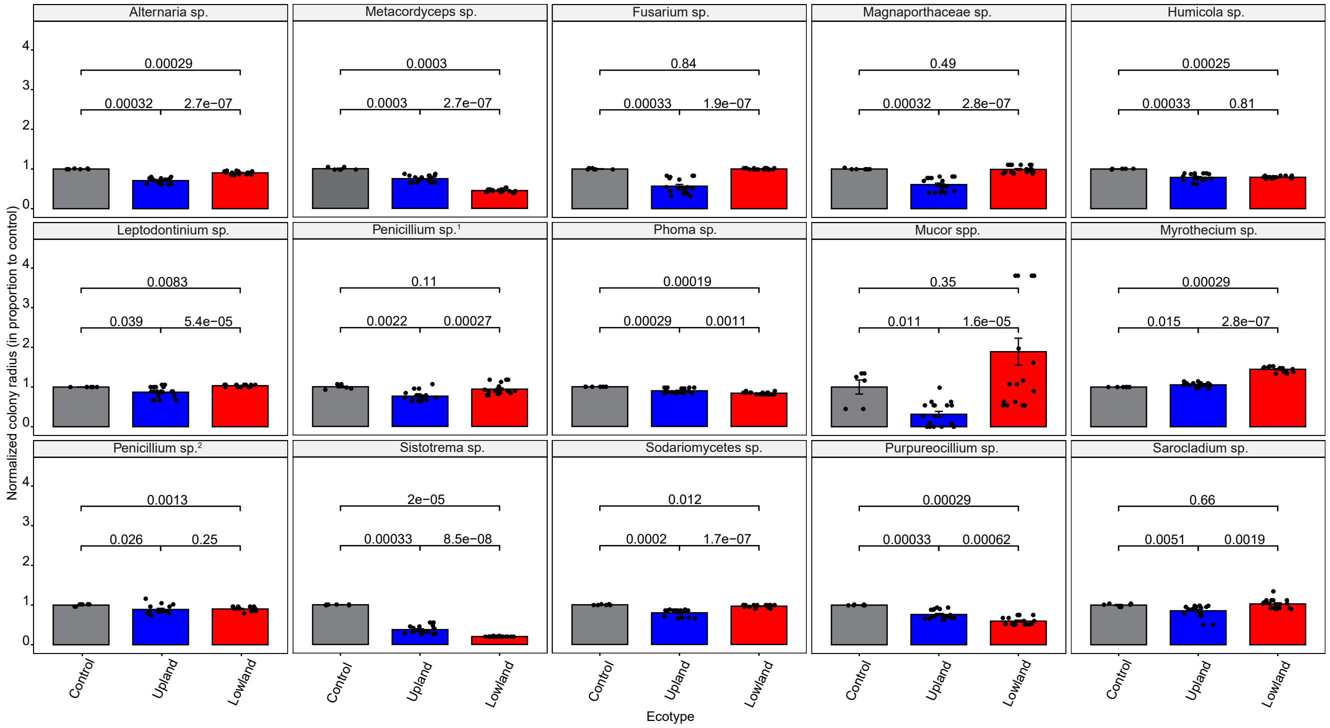

Figure S2. Effect of switchgrass root extracts on disc diffusion assay growth of 15 switchgrass rhizosphere fungal isolates. The upland switchgrass cultivars (blue) are Dacotah, Summer and Cave-in-Rock; the lowland switchgrass cultivars (red) are Alamo, Kanlow and BoMaster. The names of fungal isolates are shown on the top of each panel. The root extract concentrations were 50 mg/mL. 80% methanol was used as a negative control. The colony radiuses from the experimental groups were normalized to the control colony radiuses. n = 3 (cultivars) x 6 (replicates per cultivar) = 18 (total replicates). *Penicillium* sp.[1], GLBRC_165; *Penicillium* sp.[2], GLBRC_242 (see **Table S1** for more details).

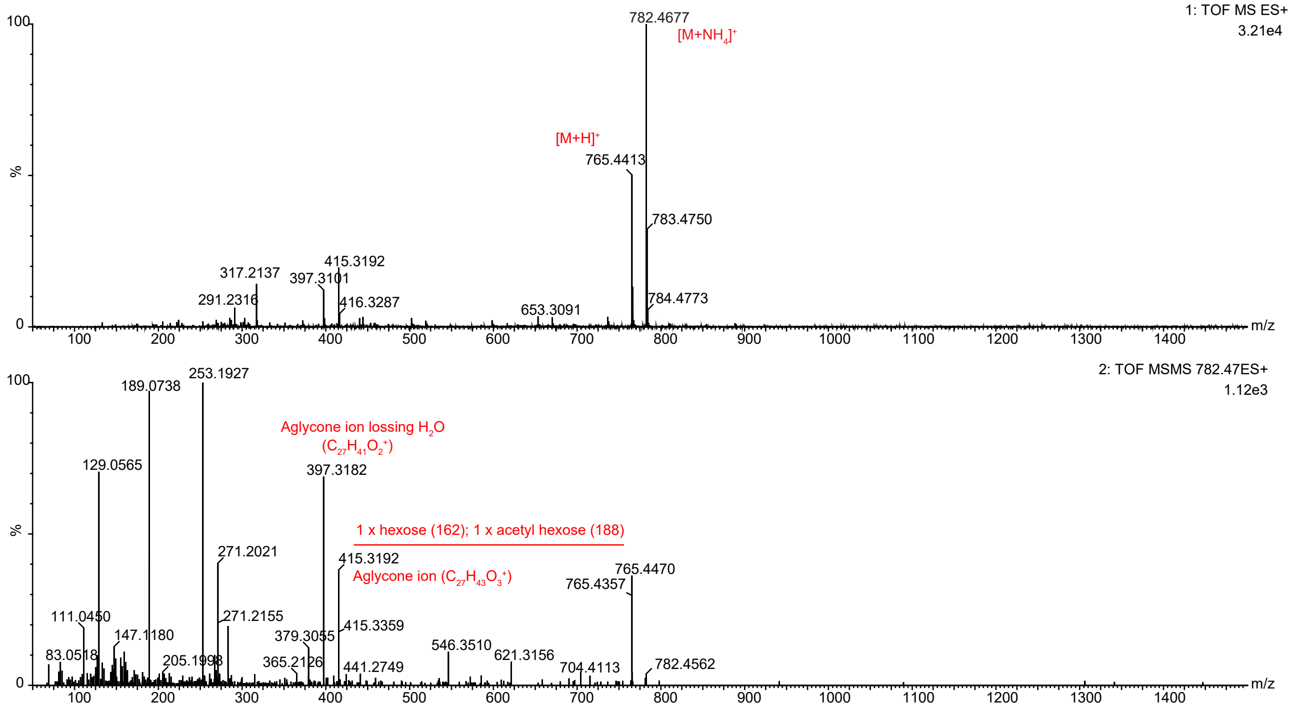

Figure S3. MS/MS spectra annotation for the saponin, SS782, obtained by positive LC-MS analysis, DDA mode. This saponin belongs to the monoglycosylated saponin class, D415, with a formular $C_{41}H_{64}O_{13}$ (**Suppl. Data 5**). It was identified by the Progenesis QI as the feature, '11.89_382.2417n' (**Suppl. Data 1 and 4**) in the positive mode LC-MS analysis. Top trace: survey scan; bottom trace: MS/MS. The molecular ions, sapogenin aglycone fragment ions and neutral mass loss are indicated. 'M' standards for molecular ion.

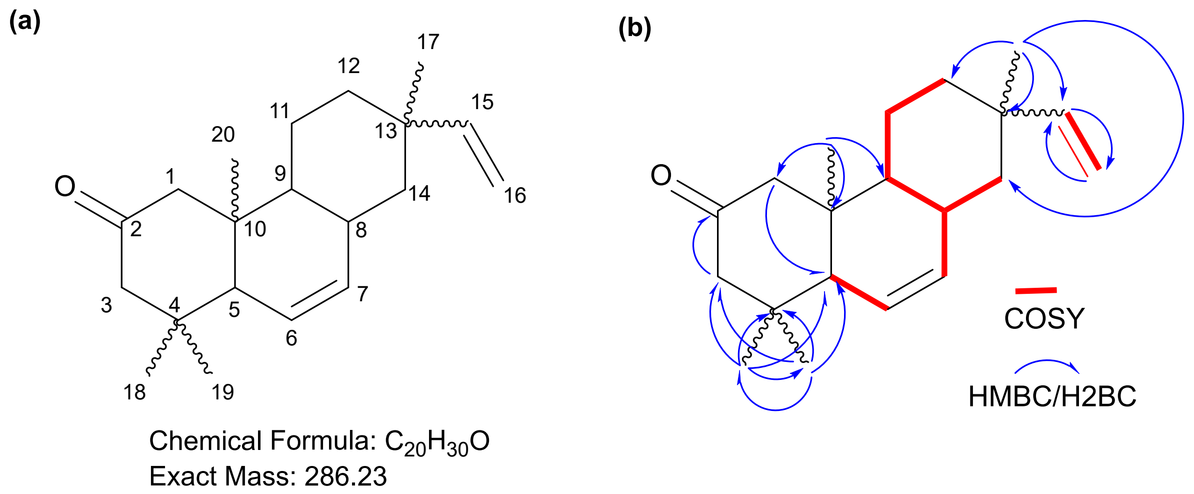

Figure S4. Structure (a) and key COSY (red, bold) and HMBC/H2BC (blue arrow) correlations (b) for the abietane diterpenoid – Di287 – purified from the switchgrass root extracts.

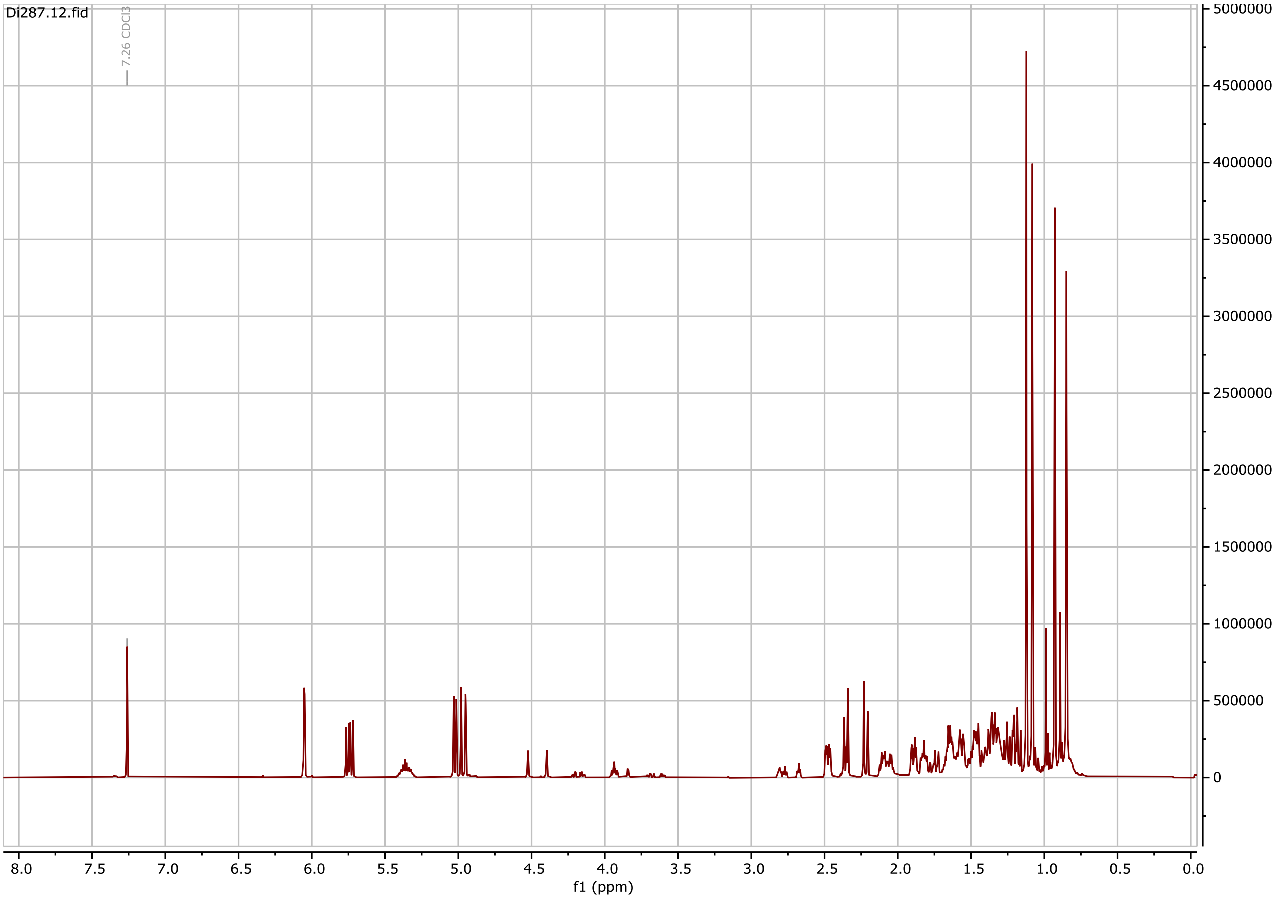

Figure S5. ^{1}**H NMR spectrum for the diterpenoid Di287.**

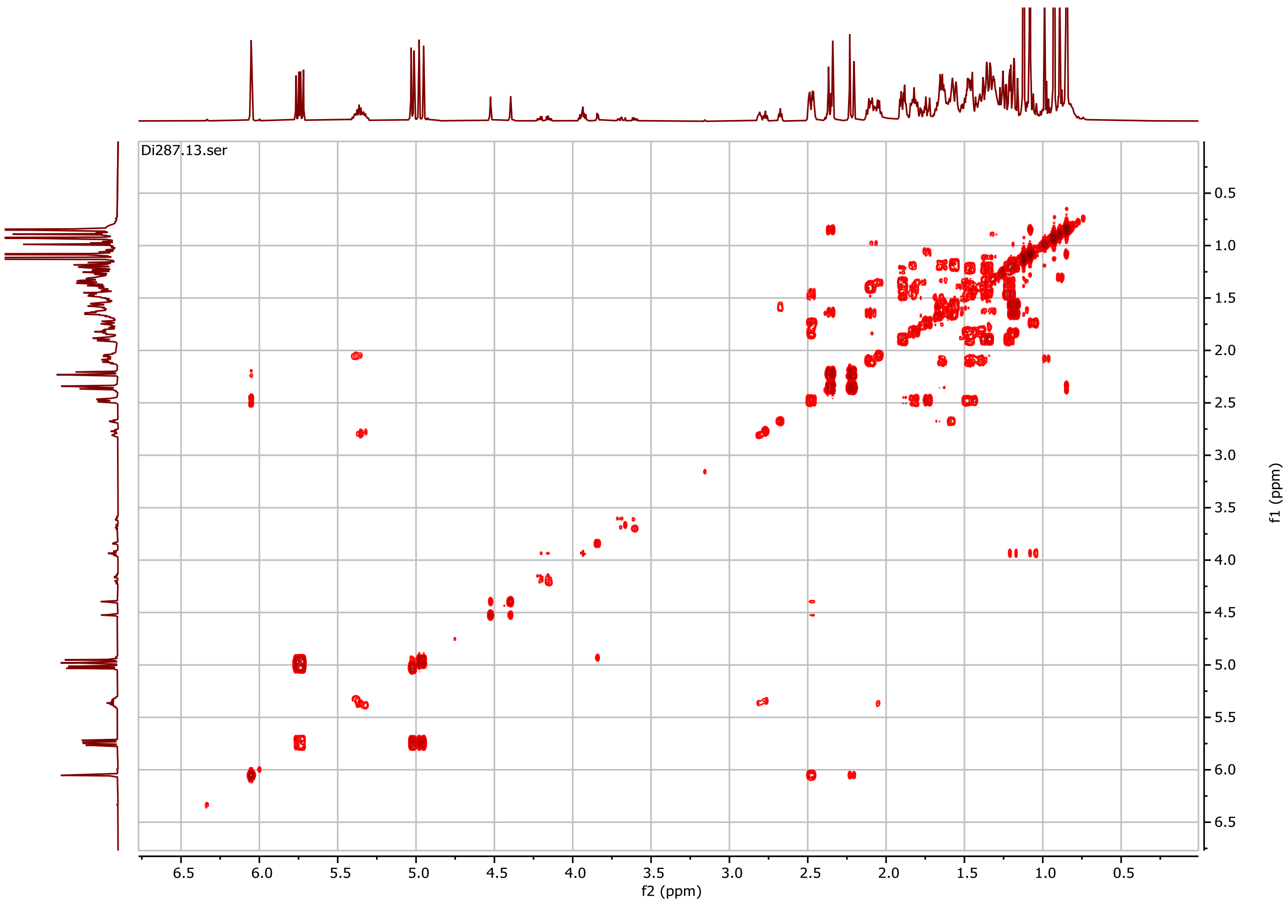

Figure S6. gCOSY ^{1}H – ^{1}H NMR spectrum for the diterpenoid Di287.

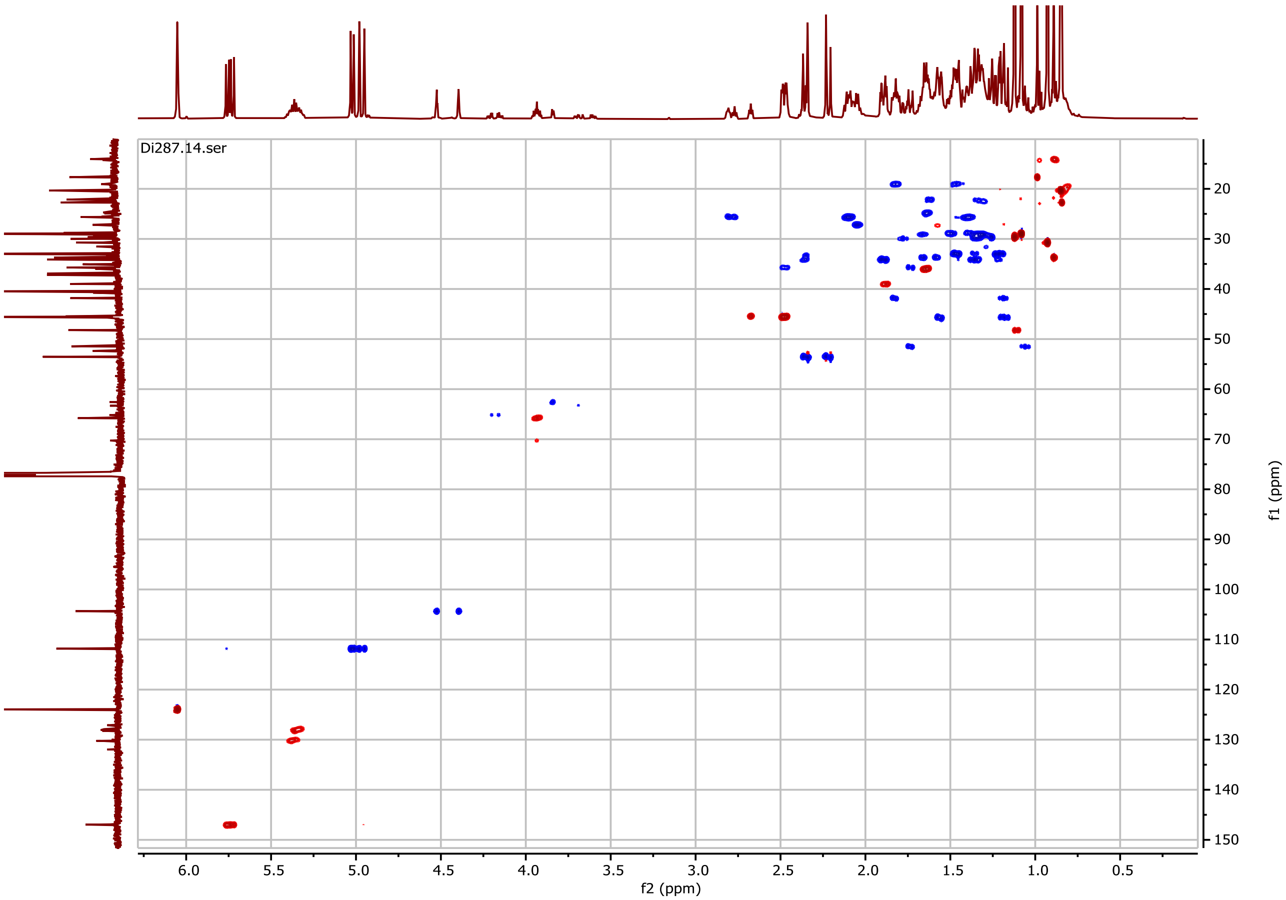

Figure S7. gHSQCAD ^{1}H – ^{13}C NMR spectrum for the diterpenoid Di287.

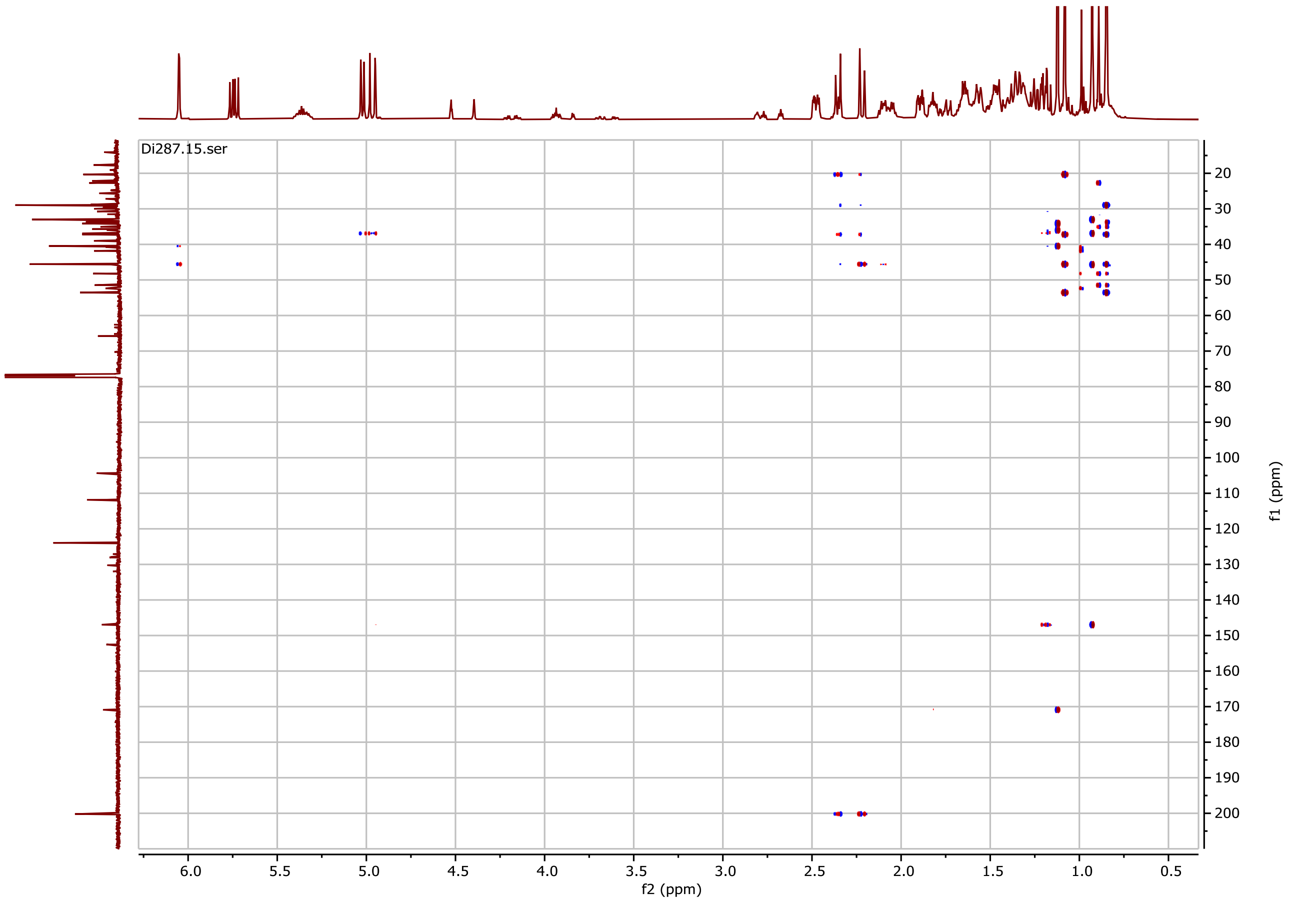

Figure S8. gHMBCAD ^{1}H – ^{13}C NMR spectrum for the diterpenoid Di287.

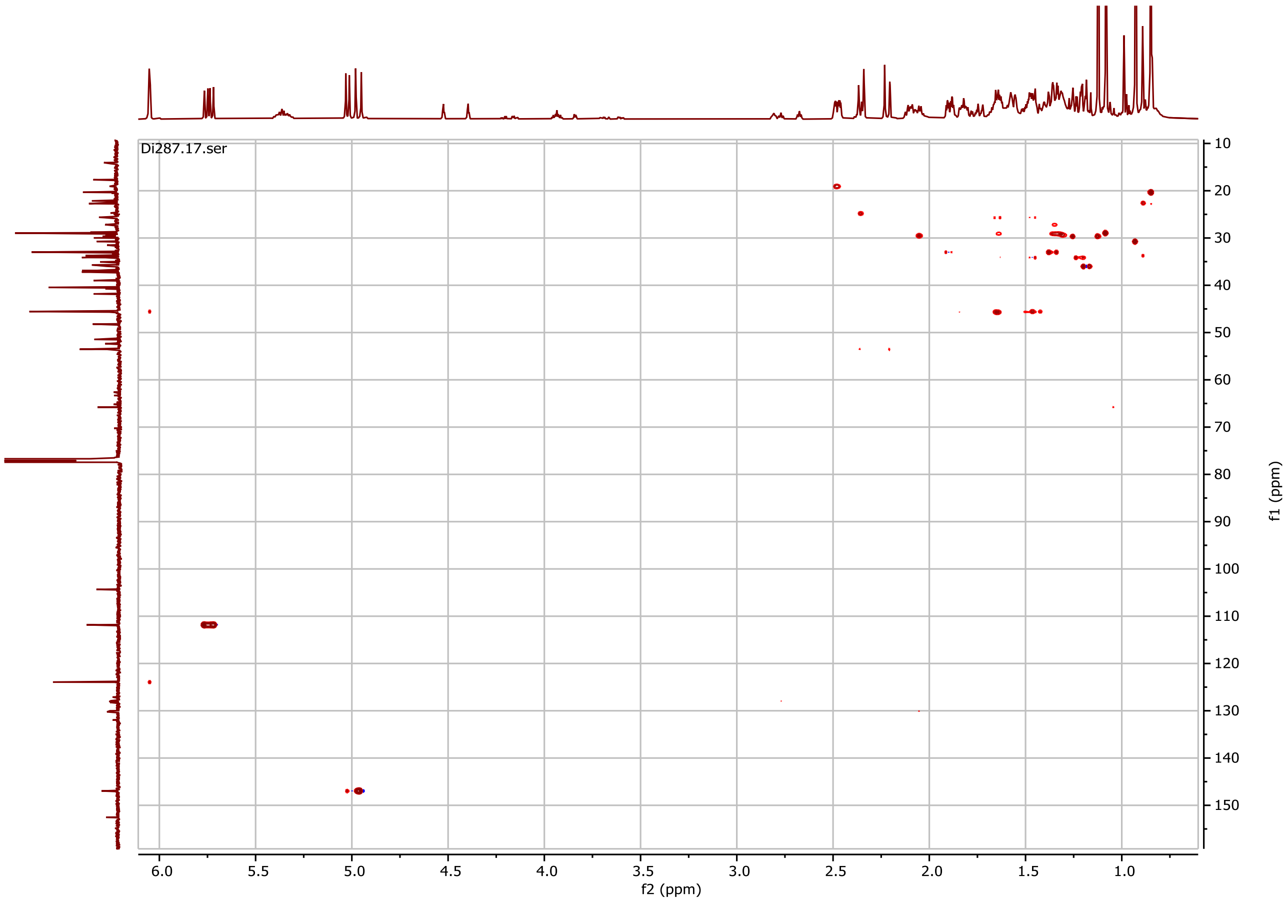

Figure S9. gH2BCAD ^{1}H – ^{13}C NMR spectrum for the diterpenoid Di287.

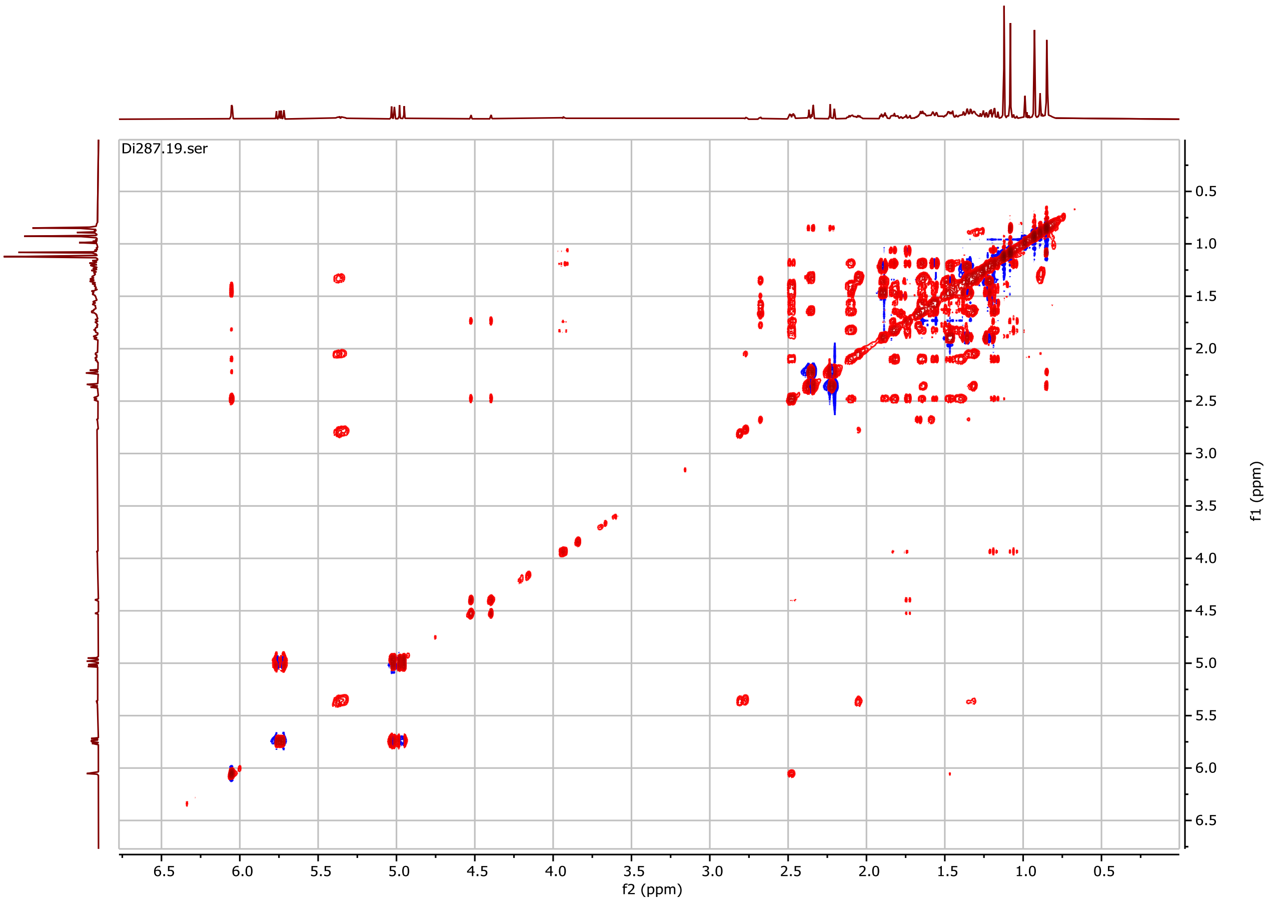

Figure S10. gTOCSY ¹H – ¹H NMR spectrum for the diterpenoid Di287.

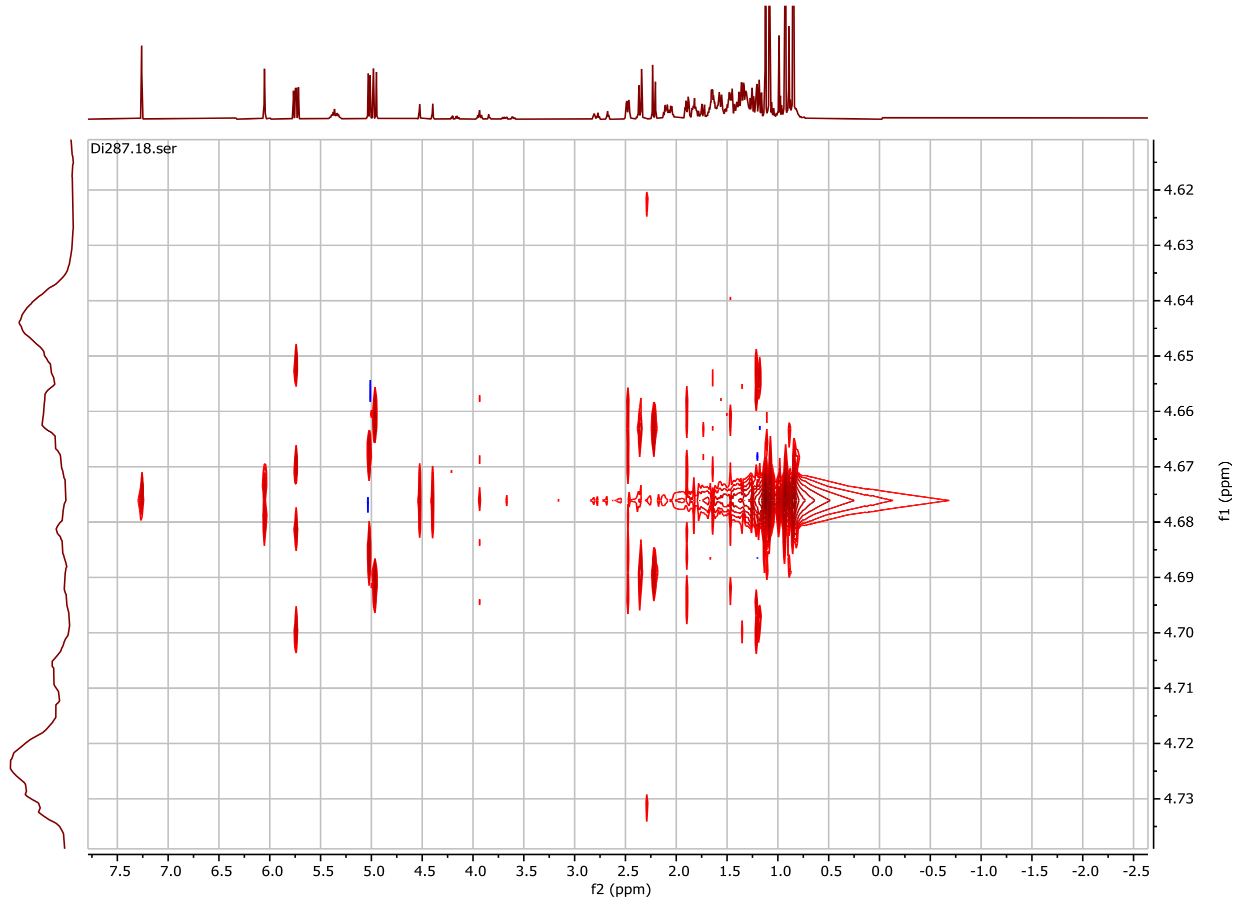

Figure S11. HOMO2DJ NMR spectrum for the diterpenoid Di287.

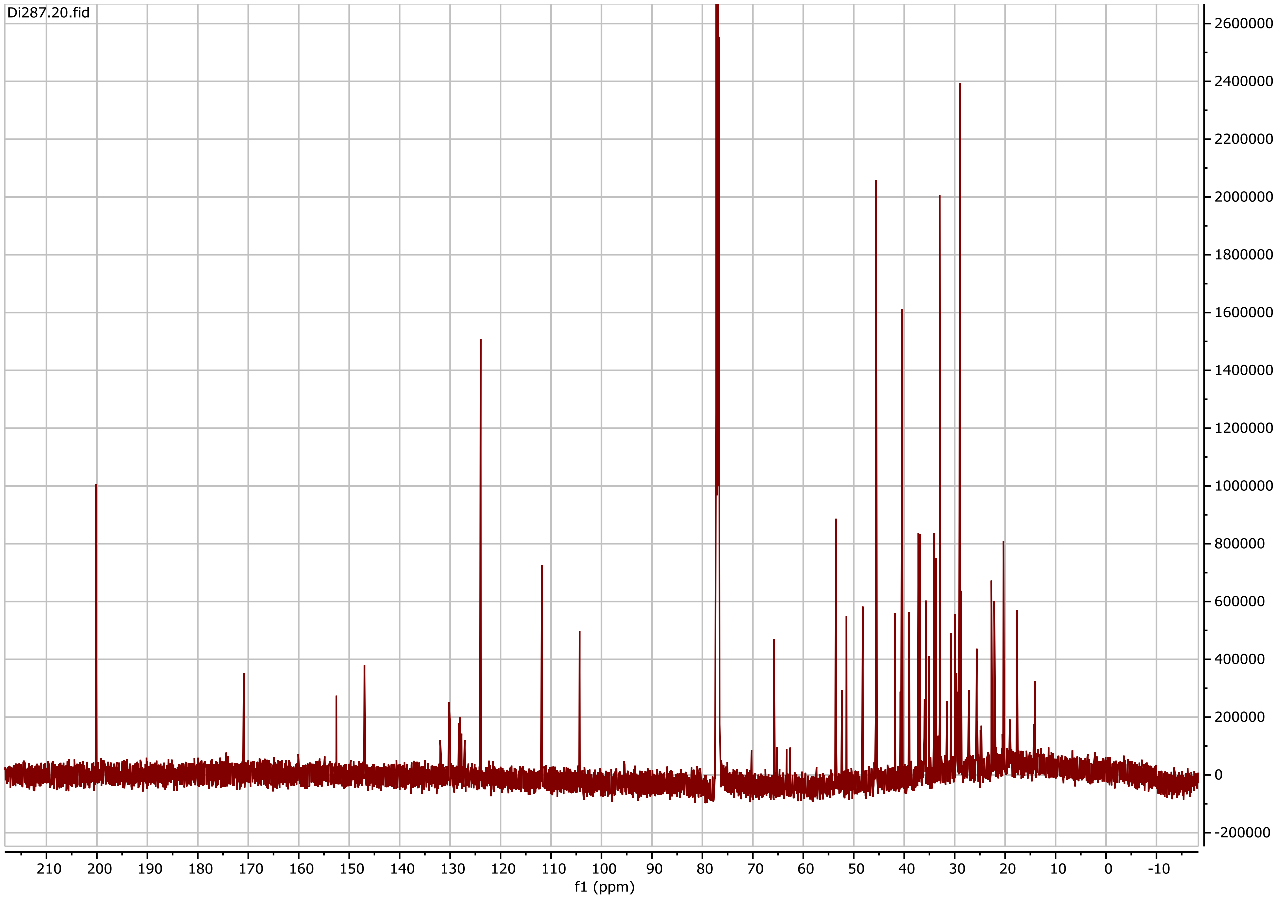

Figure S12. ^{13}C NMR spectrum for the diterpenoid Di287.

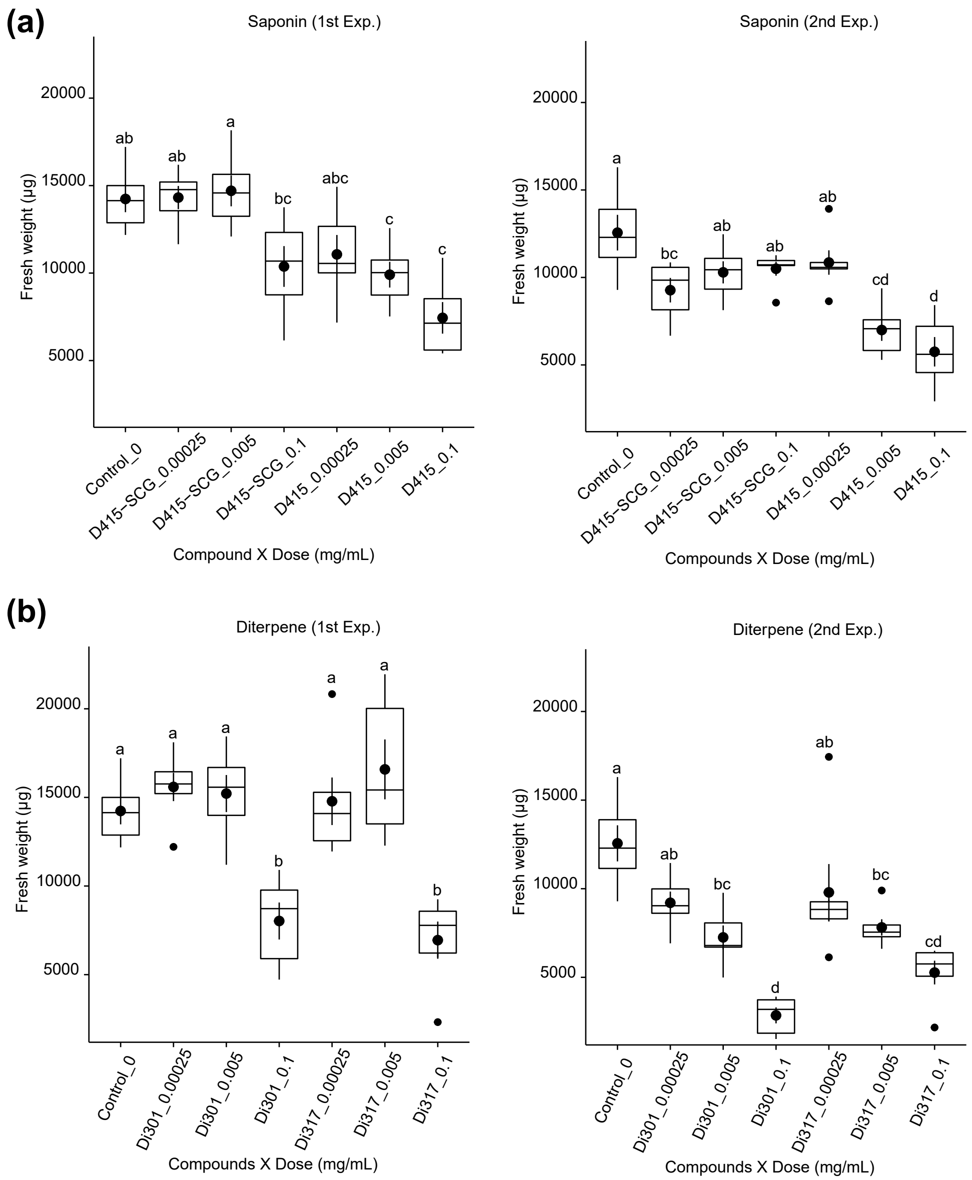

Figure S13. The saponins and diterpenoids inhibited *Linnemannia elongata* growth in liquid-based bioassays. Two saponins, D415-SCG and D415 (a), and two diterpenoids, Di301 and Di317 (b), were tested in two independent assays. The three tested concentrations, 0.01, 0.005 and 0.00025 mg/mL, for each metabolite treatment were used. The letters on top of the boxes indicate significant difference tested by ANOVA performed with Tukey's HSD test (n = 6, FDR ≤ 0.05). The lower and upper limits of the boxes represent 1st and 3rd quartile, respectively, the large dots represent means, small dots represent outlier, horizontal lines within boxes represent median, and the lower and upper vertical whiskers represent the extreme values within the 1.5 times interquartile range. 1st and 2nd represent independent replicates.

Isolate #	Taxon	Genbank Accession
GLBRC_120	*Fusarium*	OM106318
GLBRC_138	*Fusarium*	OM106336
GLBRC_165	*Penicillium*	OM106363
GLBRC_180	*Phoma*	OM106378
GLBRC_210	Sordariomycetes	OM106408
GLBRC_225	Magnaporthaceae	OM106423
GLBRC_242	*Penicillium*	OM106440
GLBRC_247	*Humicola*	OM106445
GLBRC_256	*Myrothecium*	OM106454
GLBRC_275	*Sarocladium*	OM106473
GLBRC_331	*Purpureocillium*	OM106529
GLBRC_496	*Trichoderma*	OM106692
GLBRC_573	*Alternaria*	OM106763
GLBRC_605	*Mucor*	OM106792
GLBRC_635	*Linnemannia*	OM106822
GLBRC_644	*Leptodontidium*	OM106831
GLBRC_1207	*Metarhizium*	OQ933124
GLBRC_1212	*Sistotrema*	OQ933125

Table S1. Names and Genbank accession numbers for the 18 switchgrass fungal isolates. 'GLBRC' stands for the Great Lakes Bioenergy Research Center.

Position	^{1}H δ (ppm)	^{13}C δ (ppm)	J (Hz)
1a	1.35	34.13	
1b	1.89	34.13	
2		200.19	
3a	2.34 (dd)	53.61	15.85
3b	2.2 (dd)	53.61	16.03
4		37.23	
5	2.67 (t)	45.41	
6	5.36	128.13	
7	6.05 (d)	124.03	3.28
8	2.48 (ddt)	45.58	
9	1.64	36.01	
10		40.41	
11a	1.59	33.61	
11b	1.65	33.78	
12a	1.21	32.98	
12b	1.46	33.01	
13		36.87	
14a	1.56	45.66	
14b	1.18	45.70	
15	5.74 (dd)	146.93	17.46, 11.06
16a	4.98 (dd)	111.90	10.38
16b	5.03 (dd)	111.90	17.14
17	0.93 (s)	30.71	
18	1.08 (s)	29.00	
19	0.85 (s)	20.28	
20	1.12 (s)	29.68	

Table S2. NMR chemical shift assignments of the diterpenoid Di287. Chemical formular: C20H30O
