# Supplementary material for: Anti-fungal bioactive terpenoids in the bioenergy crop switchgrass (Panicum virgatum) may contribute to ecotype-specific microbiome composition
Source: Commun Biol. 2023 Sep 7;6:917. doi: 10.1038/s42003-023-05290-3 (PMC10485007; doi:10.1038/s42003-023-05290-3)
Supplement: Supplementary file 2 — Description of Additional Supplementary Files [file 42003_2023_5290_MOESM2_ESM.pdf]

Description of Additional Supplementary Files

File name: Supplementary Data 1
Description: The untargeted metabolomics dataset.

File name: Supplementary Data 2
Description: Annotations for the metabolite features based on LC-MS/MS spectra.

File name: Supplementary Data 3
Description: The upland switchgrass ecotype differentially accumulated features (DAFs) enriched in the upland root extracts and fractions identified by OPLS-DA analysis.

File name: Supplementary Data 4
Description: The lowland switchgrass ecotype differentially accumulated features (DAFs) enriched in the lowland root extracts and fractions identified by the OPLS-DA analysis.

File name: Supplementary Data 5
Description: Metabolite annotation and documentation for the UPLC-QTOF-MS data of switchgrass steroidal saponins and diterpenoids.

File name: Supplementary Data 6
Description: The numerical source data of the antifungal activity screening for switchgrass whole root extracts in the disc diffusion bioassay (Fig 3 b - d and Fig S2).

File name: Supplementary Data 7
Description: The numerical source data of the antifungal activity screening for switchgrass root fractions against Linnemannia elongata in the disc diffusion bioassay (Fig 3 e).

File name: Supplementary Data 8
Description: The numerical source data of the antifungal activity screening for switchgrass root fractions against Trichoderma sp. in the disc diffusion bioassay (Fig 3 f).

File name: Supplementary Data 9
Description: The numerical source data of the antifungal activity screening for switchgrass root fractions against Fusarium sp. in the disc diffusion bioassay (Fig 3 g).

File name: Supplementary Data 10
Description: The numerical source data of the antifungal activity screening for the HPLC purified compounds against Linnemannia elongata in the disc diffusion bioassay (Fig 5 b and c).

File name: Supplementary Data 11
Description: The numerical source data of the antifungal activity screening for the HPLC purified compounds against Trichoderma sp. in the disc diffusion bioassay (Fig 5 b and c).

File name: Supplementary Data 12
Description: The numerical source data of the antifungal activity screening for the HPLC purified compounds against Fusarium sp. in the disc diffusion bioassay (Fig 5 b and c).

File name: Supplementary Data 13
Description: The numerical source data of the antifungal activity screening for the HPLC purified compounds against Linnemannia elongata in the liquid culture bioassay (Fig S13).
